# Transcriptional Rewiring of the Sex Determining *dmrt1* Gene Duplicate by Transposable Elements

**DOI:** 10.1371/journal.pgen.1000844

**Published:** 2010-02-12

**Authors:** Amaury Herpin, Ingo Braasch, Michael Kraeussling, Cornelia Schmidt, Eva C. Thoma, Shuhei Nakamura, Minoru Tanaka, Manfred Schartl

**Affiliations:** 1 University of Würzburg, Physiological Chemistry I, Biozentrum, Würzburg, Germany; 2 Laboratory of Molecular Genetics for Reproduction, National Institute for Basic Biology 5-1, Higashiyama, Okazaki, Japan; 3 University of Würzburg, Rudolf-Virchow-Center for Experimental Biomedicine (DFG research Center), Würzburg, Germany; Stanford University, United States of America

## Abstract

Control and coordination of eukaryotic gene expression rely on transcriptional and posttranscriptional regulatory networks. Evolutionary innovations and adaptations often require rapid changes of such networks. It has long been hypothesized that transposable elements (TE) might contribute to the rewiring of regulatory interactions. More recently it emerged that TEs might bring in ready-to-use transcription factor binding sites to create alterations to the promoters by which they were captured. A process where the gene regulatory architecture is of remarkable plasticity is sex determination. While the more downstream components of the sex determination cascades are evolutionary conserved, the master regulators can switch between groups of organisms even on the interspecies level or between populations. In the medaka fish (*Oryzias latipes*) a duplicated copy of *dmrt1*, designated *dmrt1bY* or *DMY*, on the Y chromosome was shown to be the master regulator of male development, similar to *Sry* in mammals. We found that the *dmrt1bY* gene has acquired a new feedback downregulation of its expression. Additionally, the autosomal *dmrt1a* gene is also able to regulate transcription of its duplicated paralog by binding to a unique target Dmrt1 site nested within the *dmrt1bY* proximal promoter region. We could trace back this novel regulatory element to a highly conserved sequence within a new type of TE that inserted into the upstream region of *dmrt1bY* shortly after the duplication event. Our data provide functional evidence for a role of TEs in transcriptional network rewiring for sub- and/or neo-functionalization of duplicated genes. In the particular case of *dmrt1bY*, this contributed to create new hierarchies of sex-determining genes.

## Author Summary

Evolutionary innovations and adaptations often require rapid changes in gene regulation. Transposable elements constitute the most dynamic part of eukaryotic genomes. Insertions of transposable elements can influence the expression of surrounding genes by donating new regulatory elements. A longstanding hypothesis postulates that the dispersal of transposable elements may rewire regulatory links between genes, thereby changing regulatory networks and shuffling regulatory cascades. A regulatory hierarchy of remarkable plasticity is the sex determination cascade. In the course of animal evolution, new master regulators frequently replace the sex determination gene on top of the hierarchy. In the medaka fish, a duplicate of the *dmrt1* transcription factor gene, *dmrt1bY*, has become the sex master regulator. Its ancestor *dmrt1a*, in contrast, has a downstream position in the sex determination cascade. We show that after the duplication of the *dmrt1* gene, the new hierarchy has been established by the insertion of a transposable element into the regulatory region of the *dmrt1bY* gene on the sex chromosome. This transposable element, harboring a Dmrt1 binding site, enables the self- and cross-regulation of *dmrt1bY* expression by Dmrt1 proteins. Our study therefore provides strong evidence for the important role of transposable elements in the rewiring of gene regulatory networks.

## Introduction

Control and coordination of eukaryotic gene expression rely on transcriptional and posttranscriptional regulatory networks. From an evolutionary point of view innovations and changes in given functional linkages of regulatory networks have to occur at the DNA level by alteration of the *cis*-regulatory sequence defining transcription factor binding sites. While such alterations may occur in any *cis*-regulatory module, they will have fundamentally different effects depending on where in the structure of the network they occur (see [1] for review). After the discovery of the ubiquitousness of repeated sequences, a long standing hypothesis proposed that repeated sequences were likely to be active in the 5′ regions of genes controlling transcription [2] and that they could move and supply evolutionary variations [3].

From an evolutionary perspective, transposable elements (TEs) have recently been attributed an important role in shaping the gene regulation landscape [4],[5],[6]. In spite of and, to some extent, because of their selfish and parasitic nature, the movement and accumulation of TEs have exerted a strong influence on the evolutionary trajectory of their host genome [7]. Many ways have been illustrated through which TEs can directly influence the regulation of nearby gene expression, both at the transcriptional and post-transcriptional levels (for review see [5]). Genome-scale bioinformatic analyses have shown that many promoters and polyadenylation signals of human and mouse genes are derived from primate and rodent–specific TEs respectively [8],[9]. Hence, it is postulated that insertion of TEs harbouring “ready-to-use” *cis*-regulatory sequences probably contributed to the establishment of lineage-specific patterns of gene expression [10]. In addition to donating *cis*-elements and creating new regulatory networks, the movement and accumulation of TEs have recently been proposed to participate in the rewiring of pre-established regulatory networks (see [5] for review).

Such rewiring is especially important when rapid adaptation of existing regulatory networks or new networks become necessary. One system where fast changes obviously regularly occurred during evolution is the genetic control of sexual development [11],[12]. It is well documented that different groups of organisms and sometimes even closely related species of different populations of the same species have fundamentally different modes of sex determination. Comparative evolutionary studies of the genetic cascades controlling sex determination in different species revealed that the master genes at the top of the regulatory hierarchy can change dramatically as new species evolve, while the downstream genes at the bottom of the hierarchy remain the same, exerting essentially identical functions from one species to the next (see [12],[13] for review).

The most conserved downstream component characterized to date, a gene with homology to both the *Drosophila doublesex* and *C. elegans mab-3* sex regulatory genes, is the *Dmrt1* (Doublesex and Mab-3 Related Transcription factor 1) gene of vertebrates [14]. All three genes encode proteins sharing a common DNA-binding domain and belong to the DM domain gene family, which has been shown to be involved in sex determination and differentiation in organisms as phylogenetically divergent as corals, worms, flies and all vertebrate groups ranging from fish to mammals. Of note, in humans, haploinsufficiency of the genomic region that includes *DMRT1* and its paralogs *DMRT2* and *DMRT3* leads to XY male to female sex reversal [15]. In chicken and other avian species *Dmrt1* is located on the Z chromosome, but absent from W, making it an excellent candidate for the male sex-determining gene of birds [16],[17].

In the medaka fish (*Oryzias latipes*), which has XY-XX sex determination, a duplicated copy of *dmrt1*, designated *dmrt1bY* or *DMY*, on the Y-chromosome was shown to be the master regulator of male development [18],[19], similar to *Sry* in mammals. Interestingly, also in *Xenopus laevis* a W-specific duplicate of *dmrt1* was shown to participate in primary gonad development [20]. Because medaka *dmrt1bY* acts, like *Sry*, as a dominant male determiner [21], it is believed that it is functionally equivalent to the mammalian gene and might share many molecular features [22],[23]. Although many of the early cellular and morphological events downstream of *Sry* have been characterized, as well as a number of genes involved in these processes (for review see [24]–[26]), little is known about the mode of action and the biological targets of *Sry* [27]. Interestingly, *dmrt1*, the ancestor of *dmrt1bY*, is one of these downstream effectors of *Sry*. Contrary to the situation with *Sry*, it is totally unknown how in medaka *dmrt1bY* expression is regulated. As a prerequisite to elucidate the function of *dmrt1bY*, information on its expression regulation at the transcriptional level is required. Here, we found a feed back down-regulation of the *dmrt1bY* promoter. Also *dmrt1a*, the autosomal ancestor of *dmrt1bY*, is able to down-regulate transcription of its paralog. Interestingly we found clear evidence that the major *cis*-regulatory element, pre-existing within a new medaka-specific TE at the time of its insertion, was co-opted in order to confer to Dmrt1bY its specific expression pattern after gene duplication. This is the first experimental evidence supporting a role of TEs for transcriptional network rewiring in sub- and/or neo-functionalization of duplicated genes. Additionally, in the particular case of *dmrt1bY*, this contributed to create new hierarchies of sex determining genes.

## Results

### Sequence evolution of the *dmrt1bY* promoter

To obtain insights into the sequence evolution of the *cis*-regulatory region of the *dmrt1bY* gene on the Y-chromosome, we first compared its genomic region and that of its autosomal progenitor, the *dmrt1a* gene from linkage group 9 (LG9), with those of the available *dmrt1* orthologs from other teleosts (stickleback, Tetraodon, Fugu, zebrafish), chicken and human. This phylogenetic footprinting approach should point to the conservation of regulatory elements being putatively essential for vertebrate *Dmrt1* gene expression (Figure S1). Furthermore, it could possibly indicate *cis*-regulatory subfunctionalization between the medaka *dmrt1* paralogs as observed for other pairs of duplicated genes with spatio-temporal expression divergence [28],[29]. However, our VISTA plots (Figure S1) did not reveal sequence conservation in the promoter regions of neither *dmrt1bY* nor *dmrt1a* with other vertebrates except for stretches corresponding to the teleost *MHCL* gene, which are pseudogenes in both medaka *dmrt1* 5′ regions [30]. Conserved non-coding elements were also not found between the other vertebrate sequences suggesting that the regulatory regions of vertebrate *Dmrt1* orthologs diverged strongly despite their conserved position in the sex determination cascade. High turn-over of *cis*-regulatory regions in the face of conserved expression is commonly found for vertebrate genes [31].

**Figure 1:**
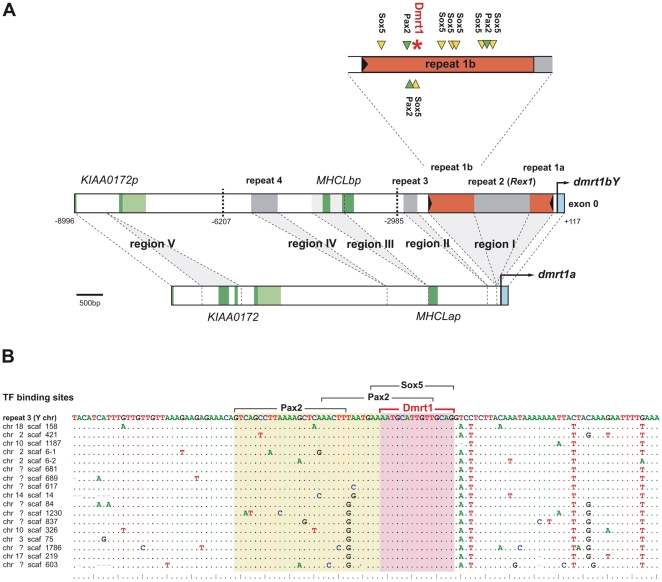
Comparative analysis of the *dmrt1bY* promoter and its transcription factor binding sites. (A) The analyzed promoter region of *dmrt1bY* in comparison to its *dmrt1a* paralog. Length differences between the promoters are based on regions I–IV, of which three (I, II, IV) have been added to the *dmrt1bY* promoter after duplication, while two others have been lost from the *dmrt1bY* promoter (*KIAA0172* region V) or from the *dmrt1a* promoter (*MHCLp* region III), respectively. Bold dashed lines indicate cutting sites for transcriptional regulation analyses. Region I contains a putative *Izanagi* DNA transposons (repeat 1), into which a *Rex1* element (repeat 2) was inserted secondarily. The upstream part of split repeat 1 (repeat 1b) in the *dmrt1bY* promoter contains multiple Sox5 and Pax2 binding sites as well as a Dmrt1 binding site. Dark green indicates coding sequence of genes and pseudogenes, light green indicates their untranslated regions. (B) Alignment of the Y chromosomal repeat 1b with 17 exemplary repeat copies in the medaka genome. Dots indicate conserved sites. The Dmrt1 binding site is perfectly conserved suggesting that it is an integral part of repeat 1 elements. All 28 copies of repeat 1b in medaka with the Dmrt1 binding sites are listed in Table S1.

However, longer stretches of conservation between promoter regions of *dmrt1bY* and *dmrt1a* in medaka were evident (Figure S1). Thus, we compared in more detail the promoter regions of the medaka *dmrt1* paralogs upstream of the transcriptional start site to the last exons of their upstream gene, *KIAA00172*, which is a pseudogene on the Y chromosome but functional on the autosomal LG9 [30] (Figure 1A). This region spans around 9 Kb on the Y chromosome but only around 6 Kb on autosomal LG9.

In the upstream sequence of *dmrt1* paralogs, five regions contribute to length divergence between autosome and Y chromosome (Table 1, Figure 1A, Figure S1 and Figure S2). Region I located 69 bp upstream of the transcriptional start site of *dmrt1bY* is over 2 Kb in length and absent from *dmrt1a*. Similarly, regions II–IV further upstream are only found on the Y chromosome but not on the autosome.

**Table 1:** Characterization of regions contributing to length differences between *dmrt1* upstream sequences.

Region	Absent from	Repeat/Gene	Length (bp)	TF binding sites	Genomic copies^b^
**region I**	*dmrt1a*		2,348		
		**repeat 1 (** ***Izanagi*** **)** a	1,316		13
		repeat 1a	440	Pax2, HMG-A	
		repeat 1b	876	Sox5 (7x), Pax2 (2x), **Dmrt1**	
		**repeat 2 (** ***Rex1*** **)** a	1,024	Sox5, Pax2, Est Rec, Sox9 (2x), Prog Rec, And Rec	14
**region II**	*dmrt1a*	**repeat 3**	315	-	49
**region III**	*dmrt1a*	***MHCLp***	598	Sox9, HMG-P1, WT1	1
**region IV**	*dmrt1a*	**repeat 4**	496	And Rec (2x), SF1, Pax2	68
**region V**	*dmrt1bY*	***KIAA0172***	728	HGM-A, HGM-P1	1

Abbreviations: TF: transcription factor.

a Repeat 2 insertion splits repeat 1 into repeat 1a and repeat 1b.

b Copy numbers in the medaka genome assembly (version HdrR, Oct 2005) estimated by BLASTN searches with ≥85% sequence identity over ≥85% of query length.

Region V, in contrast, is missing from the Y chromosome but present on the autosome. This region contains two exons of the *KIAA0172* gene and obviously has been lost during the pseudogenization of the Y chromosomal *KIAA0172* copy after the duplication of the *dmrt1* region [30].

Region III is directly adjacent to the *MHCL* pseudogenes [30],[32] present in both *dmrt1* promoters and a stretch of sequence similarity with other teleost *MHCL* orthologs is found within region III on the Y chromosome (Figure S1). Furthermore, region III is present only once in the medaka genome and does not constitute a repetitive element according to RepeatMasker analysis. Thus, region III seems to have been lost from the autosomal *dmrt1a* region after duplication, but has been kept on the Y chromosome.

Next, we further characterized the additional regions I, II and IV for the presence of repetitive sequences. As each of these three regions is present in multiple copies in the medaka genome (Table 1), they constitute repetitive sequences that were rather added to the Y chromosomal region than lost from the autosome. Each repeat element is only present once in the analyzed section of the *dmrt1bY* region and is often found in poorly assembled regions of the medaka genome emphasizing their repetitive character. BLAST searches in other teleost genomes failed to find the repeat elements identified here in other species suggesting that they are specific to the medaka lineage.

Particularly, region I is subdivided into three parts with a non-LTR retrotransposable *Rex1* element of the LINE family (class I transposable elements) as middle segment (“repeat 2”) (Table 1).

The surrounding parts (“repeat 1a”, “repeat 1b”) also represent repetitive sequences with multiple copies in the medaka genome. These two repeat regions are found side by side in other regions of the genome. Thus, they together build a larger repeat element (“repeat 1”) into which repeat 2 was inserted (see below). Repeat 1 has a length of 1316 bp and is characterized by 27 bp terminal inverted repeats (TIRs) (5′-CAATGAGTTATATCACTAGAGGAGACA-3′) assigning it to DNA transposons (class II transposable elements). However, it does not contain a transposase gene or any other open reading frame and thus constitutes a non-autonomous class II element. Only few diagnostic motifs are available to classify such elements [33]. Repeat 1 in the *dmrt1bY* promoter has a 8 bp target site duplication (5′- GTGTGGCT-3′) and other copies of this element in the medaka genome have target site duplications of the same length. Repeat 1 is found in multiple copies in the medaka genome (Table 1 and Table S1), which generally have target site duplications. This points to an active state of repeat 1 in the medaka genome.

From the consensus sequence of the multiple repeat 1 elements in the medaka genome, a THAP protein domain composed of three putative exons was deduced (Figure S2 and Figure S3). In the repeat 1 element in the *dmrt1bY* promoter, the second putative exon of the THAP domain has been disrupted by the insertion of the repeat element (Figure S2 and Figure S3). The THAP domain is a DNA-binding zinc finger motif present in the *P* element transposases from *Drosophila* [34]. Furthermore, the terminal motif of repeat 1 is similar to the consensus sequence for the *P* element superfamily of DNA transposons (5′-YARNG-3′) [7]. Thus, we conclude that we have identified a new, medaka-specific non-autonomous *P* element element that we term *Izanagi* (named after an ancient Japanese deity, “*the male who invites*”; for etymology see Text S1).

Vertebrate mobile DNA transposons of the *P* element family have been only found so far in zebrafish [35],[36]. However, the THAP domain has been recurrently recruited from domesticated *P* elements during chordate evolution [37]. In the *Izanagi* family, the THAP domain is degenerated and, in the case of repeat 1, has been additionally disrupted by the repeat 2 insertion.

Region II (“repeat 3”) and region IV (“repeat 4”) also have multiple copies in the medaka genome (Table 1). They do not contain open reading frames and, like repeat 1, they also lack similarity to known transposable elements. Furthermore, target site duplication or other diagnostic features could not be recognized preventing further classification as putative transposable elements.

### Identification of putative transcription factor binding sites within *dmrt1bY* promoter and transcriptional activity in different cell lines

The sequence of the medaka *dmrt1bY* promoter region (9.107 Kb) was next analyzed for the presence of putative transcription factor binding sites using the MatInspector program (Figure 1A and Figure S4 and Table 1). Most interestingly, the *Izanagi* element is characterized by an overrepresentation of putative binding sites for Sox5 (Figure 1 and Figure S4). In this region seven Sox5 binding sites are present while random prediction would expect 15 times less (only 0.46 sites; MatInspector). This, together with the fact that Sox5 expression has been correlated with direct *dmrt1* promoter down-regulation in zebrafish [38] suggests that region to be of primary interest for medaka Dmrt1bY transcriptional regulation, but remains to be investigated for the proposed functional role of Sox5.

Additionally, the 9 Kb *dmrt1bY* promoter region contains several other putative transcription factor binding sites such as Pax2, HMG-box protein 1, HMG-A, Sox9, WT1 and SF1 binding sites that are reasonable candidates for gonadal-specific transcriptional regulation (Figure 1A and Figure S4 and Table 1). Several of them are conserved with the *dmrt1a* promoter (Figure S4) and might be essentially required for *dmrt1* expression.

To evaluate the mechanisms regulating *dmrt1bY* transcription, a portion of the medaka gene from +117 bp to −8990 bp of the transcriptional start site was cloned upstream of the *Gaussia* luciferase gene (pBSII-ISceI::9 Kb Dmrt1bY prom::GLuc) and the activity of the promoter was measured in a variety of cell types using transient transfection analysis. Sequential deletions of the 9 Kb promoter were generated from pBSII-ISceI::9 Kb Dmrt1bY prom::GLuc. In all three cell types basal promoter activity was detectable when using the 3 Kb proximal region (Figure S5). In fibroblast cell lines (*Xiphophorus* A2 and medaka HN2), but not in Sertoli TM4 cells, a dramatic drop in promoter activity was observed when the region from bp −2985 to −6207 was added (Figure S5). Similarly, the same decrease of promoter activity was apparent in Sertoli TM4 cells when the bp −6207 to −8996 region was additionally inserted (Figure S5). This indicates the possible presence of Sertoli cell specific transcriptional repressing sequence(s) within the most distal part of the *dmrt1bY* promoter. The most proximal part of the promoter always accounted for the basal activity in all the cell lines tested. Interestingly, two adjacent binding sites for Steroidogenic factor 1 (Sf1) are located at positions −5933 and −5524 (Figure S4). Being specifically expressed in Sertoli -and Leydig- cells, it is tempting to assume that the presence of these two distinct SF-1 binding sites nested in this −3 to −6 Kb fragment is accounting for this difference. A similar situation has been shown for the porcine *Sry* promoter for which SF-1 transactivation occurs at two SF1 binding sites [39].

### A unique Dmrt1 binding site is present in medaka *dmrt1bY* but not in *dmrt1* promoter

Using the vertebrate Dmrt1 binding site matrix [40], different *dmrt1* promoters -including medaka *dmrt1a* and *dmrt1bY*- (up to 9 KB upstream the ORF) were scanned for such target site sequences. A unique and robust Dmrt1 binding site of high prediction probability was found only in the medaka *dmrt1bY* promoter (CTGCAACAATGCATT; weight: 8.5, pValue: 1.0 e-05, lnPval:−11.492) (Figure 1A and Figures S2, S3, S4) but not in the *dmrt1a* promoter (lower threshold set to 0). Interestingly, this predicted Dmrt1 binding site is nested within the above newly described *Oryzias latipes Izanagi* element in the proximal active part of *dmrt1bY* promoter (Figure 1A and Figure S5). The medaka putative Dmrt1 binding site is present at position −2132 within repeat 1b in close proximity to the Sox5 binding site-rich region (Figure 1A and Figure S4). We first asked about the origin of this Dmrt1 binding site. It might have either evolved *de novo* from sequence provided by repeat 1b or been an integral part of such repeats and then was inserted into the *dmrt1bY* promoter after duplication. We therefore blasted the region approximately 300 bp up-and downstream of the Dmrt1 binding site to the medaka genome and aligned the obtained repeat sequences (Figure 1B). In total, we identified 28 elements that are highly similar to repeat 1b and that contain the same Dmrt1 binding site found in the *dmrt1bY* promoter (Table S1). Furthermore, the predicted Dmrt1 binding site is present in the derived *Izanagi* consensus sequence. Hence, this putative Dmrt1 binding site is a regular and conserved part of the *Izanagi* transposon family.

### Timing of the *Izanagi* element insertion into the dmrt1bY promoter

Given that the Dmrt1 binding site donated to the *dmrt1bY* promoter by the *Izanagi* element has been important for the evolution of *dmrt1bY* function within the sex determining cascade, we asked about the timing of the *Izanagi* insertion in relation to the duplication of the medaka *dmrt1* genes. The *dmrt1* gene duplication occurred in a common ancestor of medaka (*O. latipes*), *O. curvinotus* and *O. luzonensis* around 10 million years ago [41].

First, we estimated the sequence divergence between repeat 1 from the *dmrt1bY* promoter and the *Izanagi* element consensus and mapped it onto a linearized neighbour joining (NJ) tree of *dmrt1* genes from the genus *Oryzias*, which was based on neutral sites only (third codon positions). This analysis showed that the repeat 1 insertion occurred after the split from *O. mekongensis* but before the divergence of medaka, *O. curvinotus* and *O. luzonensis* (Figure S6A). This is exactly the branch on which the *dmrt1* duplication occurred. We also estimated the *dmrt1* duplication by the same method. There has to be a note of caution with dating the age of the *dmrt1* duplication due to the enhanced rate of molecular evolution of *dmrt1bY* after duplication [41]. Nevertheless, based on sequence divergence data the insertion of repeat 1 is certainly estimated to be younger than the *dmrt1* duplication (Figure S6A). Using a different nuclear marker to date the divergence of the *Oryzias* species, the *tyrosinase a* gene, a similar result was obtained (Figure S6A). The analogous analysis for the secondary insertion of repeat 2 into repeat 1, in contrast, revealed that this insertion is quite young and must have occurred in *Oryzias latipes*.

We conclude that our sequence divergence estimates are consistent with an insertion of repeat 1 and thereby of the Dmrt1 binding site shortly after the *dmrt1* duplication, supporting its importance for the evolution the Dmrt1bY sex determinator function.

### Transcription of *dmrt1bY* is regulated by its own gene product and by that of its paralog

#### 1-Dmrt1bY that down-regulates activity of its own promoter

**Figure 2:**
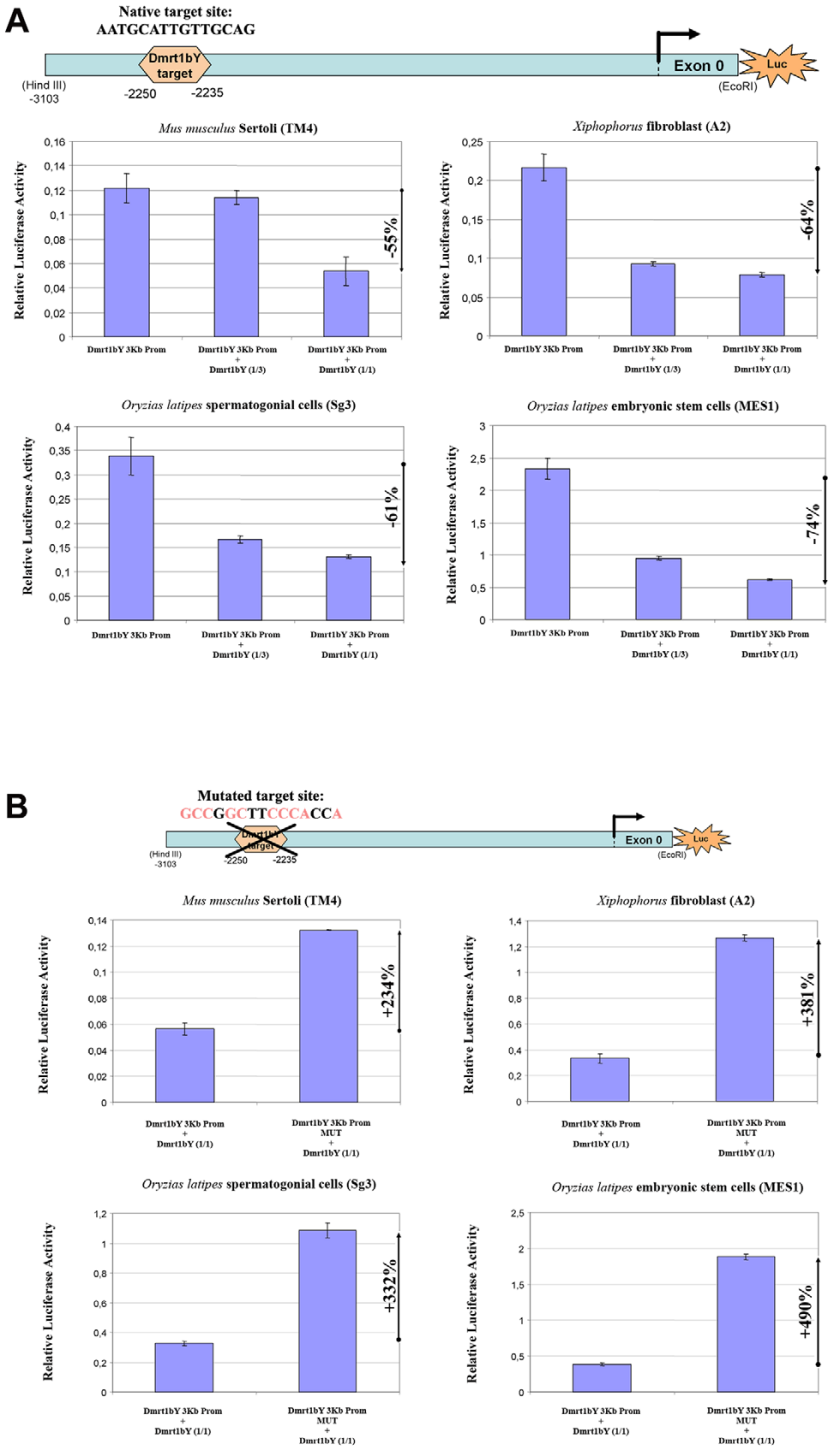
Transient-transfection analysis. (A) Transient-transfection analysis of proximal *dmrt1bY* promoter activity co-transfected in different ratios of a *dmrt1bY* expressing plasmid. The 3 Kb proximal *dmrt1bY* promoter construct was co-transfected with different amounts of *dmrt1bY*-expressing plasmid (1∶3 and 1∶1 ratios) in different cell lines. (B) Transient-transfection analysis of mutant proximal *dmrt1bY* promoter activity. In all the cell lines, when overexpressing Dmrt1bY, the 3 Kb mutant proximal *dmrt1bY* promoter construct (lacking the Dmrt1 binding site) shows higher activity compared to the “wild-type” construct containing the Dmrt1 target site. The data are presented as the firefly/Gaussia luciferase activity. Transfections were done three times; error bars represent the standard errors of the means.

Co-transfection analyses were used to examine the predicted interaction between medaka Dmrt1bY and its own promoter (Figure 2A). For this purpose, the proximal 2868 bp *dmrt1bY* promoter region containing the putative Dmrt1 binding site at position −2132 (see Figure 1 and Figure 2) fused to luciferase was used and co-transfected with different amounts of a plasmid expressing *dmrt1bY* (Figure 2A). In presence of *dmrt1bY* expressing plasmids *dmrt1bY* promoter activity was considerably reduced, up to 74%, in all cell types tested (*Mus musculus* Sertoli TM4, *Xiphophorus xiphidium* fibroblast A2 and *Oryzias latipes* spermatogonial (Sg3) and embryonic stem (MES1) cells) (Figure 2A). To confirm the possible direct interaction with the putative Dmrt1 target binding site, a *dmrt1bY* mutant promoter with a modified Dmrt1 target site was created (Figure 2B and Materials and Methods). When co-transfected with the *dmrt1bY* expressing plasmid, the activity of the mutant promoter was clearly increased (up to almost 5 fold) in comparison to the wild-type promoter (Figure 2B) whereas the mutant promoter did not reveal any significant regulation by Dmrt1bY (Figure S7).

#### 2-Dmrt1a, the autosomal ancestor of Dmrt1bY, regulates the transcriptional activity of the *dmrt1bY* promoter

**Figure 3:**
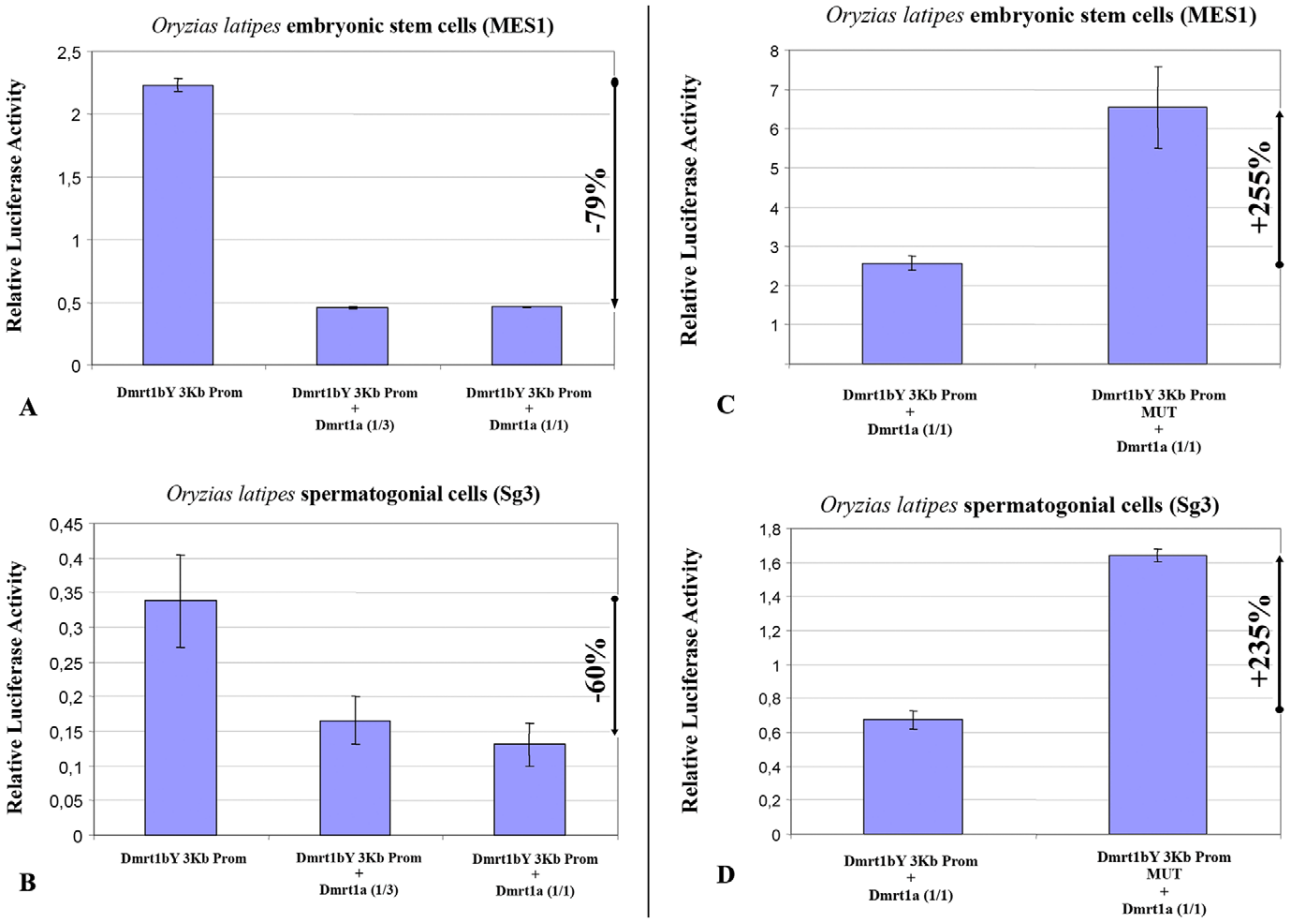
Transient-transfection analysis of *dmrt1bY* promoter activity when co-transfected with different ratios of a Dmrt1a expressing plasmid. (A,B) The 3 Kb proximal *dmrt1bY* promoter construct was co-transfected with different amounts of *dmrt1a*-expressing plasmid (1∶3 and 1∶1 ratios). (C,D) The 3 Kb mutant proximal *dmrt1bY* promoter construct was co-transfected with different amounts of *dmrt1a*-expressing plasmid (1∶3 and 1∶1 ratios). The data are presented as the firefly/Gaussia luciferase activity. Transfections were done three times; error bars represent the standard errors of the means.

We next addressed the question of a possible cross-regulation of the Dmrt1a protein towards the *dmrt1bY* promoter. The above-described experiments employing Dmrt1bY were repeated, this time using the autosomal Dmrt1a. When the proximal 2868 bp *dmrt1bY* promoter region containing the putative Dmrt1 binding site fused to luciferase was co-transfected with a plasmid expressing Dmrt1a, *dmrt1bY* promoter activity was considerably reduced –up to 92%- (Figure 3A and 3B). This reduction is higher than observed for *dmrt1bY* (ranging from −55% to 74%). Using the mutant *dmrt1bY* promoter revealed that removing the Dmrt1 target site was able to restore transcriptional activity (Figure 3C and 3D) in medaka MES1 and Sg3 cell lines.

#### 3-Dmrt1bY and Dmrt1a both bind to the putative Dmrt1 response element within *dmrt1bY* promoter

**Figure 4:**
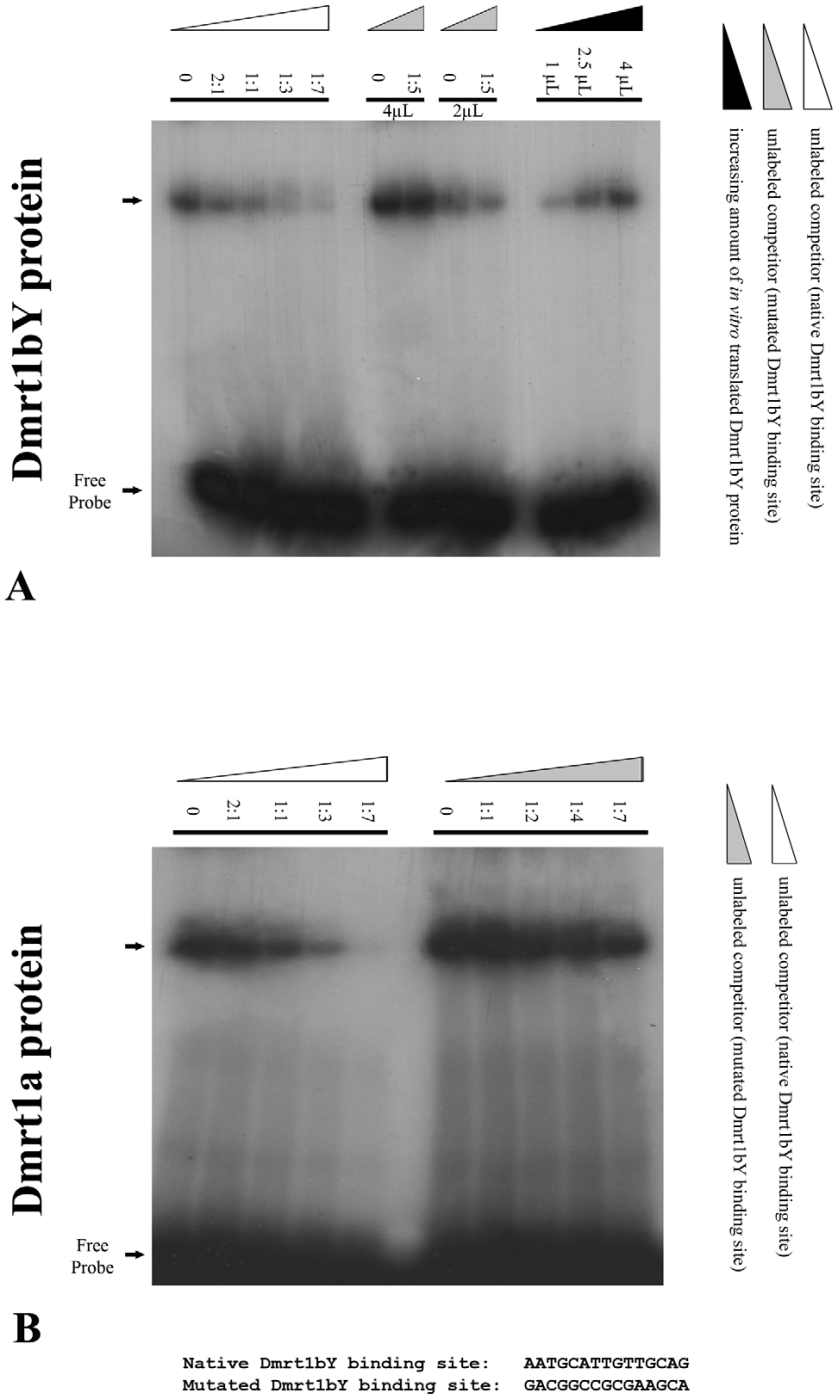
Electrophoretic Mobility Shift Analysis (EMSA) of *in vitro* translated Dmrt1bY protein interaction with the Dmrt1 binding target derived from its own promoter. Gel mobility shift using increasing amounts of either *in vitro* translated Dmrt1bY or Dmrt1a proteins to shift a constant amount of radiolabelled *dmrt1* probe. Binding reactions were resolved on a 5% polyacrylamide gel. (A) *In vitro* translated Dmrt1bY protein was incubated with radiolabelled Dmrt1bY target sequence as a probe, and non-radiolabelled probe was used as a competitor. (B) *In vitro* translated Dmrt1a protein was incubated with radiolabelled Dmrt1bY target sequence as a probe, and non-radiolabelled control (non-specific) probe was used as a competitor.

Electrophoretic mobility shift assays (EMSAs) were performed to show the direct interaction of Dmrt1a and Dmrt1bY proteins within the target site in the *dmrt1bY* promoter (Figure 4). DNA binding assays using the *dmrt1bY* Dmrt1-target sequence and *in vitro* translated Dmrt1a or Dmrt1bY demonstrated that both proteins are indeed able to bind to the Dmrt1 target sequence (position −2132 bp) (Figure 4). Binding specificity was confirmed using a mutated Dmrt1 binding site as competitor (Figure 4).

### Analysis of *dmrt1bY* expression *in vivo*

Thus far we could show feed back down-regulation of *dmrt1bY* and regulation by its paralog Dmrt1a *in vitro*. We next addressed whether this regulation indeed exists *in vivo*.

#### *In vivo* quantification of the 9Kb*dmrt1bY*promoter activity in transgenic fish indicates a strong Dmrt1a cross-regulation

**Figure 5:**
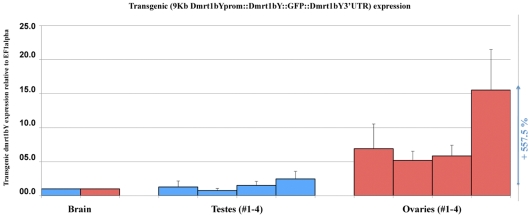
*In vivo* quantification of the 9KbDmrt1bYpromoter activity in transgenic fish. Real-time PCR quantification of the 9KbDmrt1bYpromoter activity in two different gonadal backgrounds either expressing dmrt1a (testes, #1–4) or not (ovaries, #1–4) reveals higher activity (+557.5% expression) in absence of *dmrt1a* expression. *Dmrt1bY* expression is relative to elongation factor 1 alpha (ef1 alpha) and normalised to brain background expression (set to 1).

To get a first information of the *in vivo dmrt1bY* transcriptional regulation, a transgenic line where the *dmrt1bY* promoter drives dmrt1bY::GFP fusion protein expression was established with the goal of quantifying gonadal *dmrt1bY* promoter activity in either Dmrt1a expressing or non-expressing tissues (Figure 5). These transgenic fish showed a strong correlation of higher gonadal *dmrt1bY* promoter activity (5.5 times more in average) in absence of *dmrt1a* expression (ovary) compared to the *dmrt1a* expressing (testes) background (Figure 5).

#### *In vivo* Dmrt1bY binding to the *Izanagi* Dmrt1 target site

**Figure 6:**
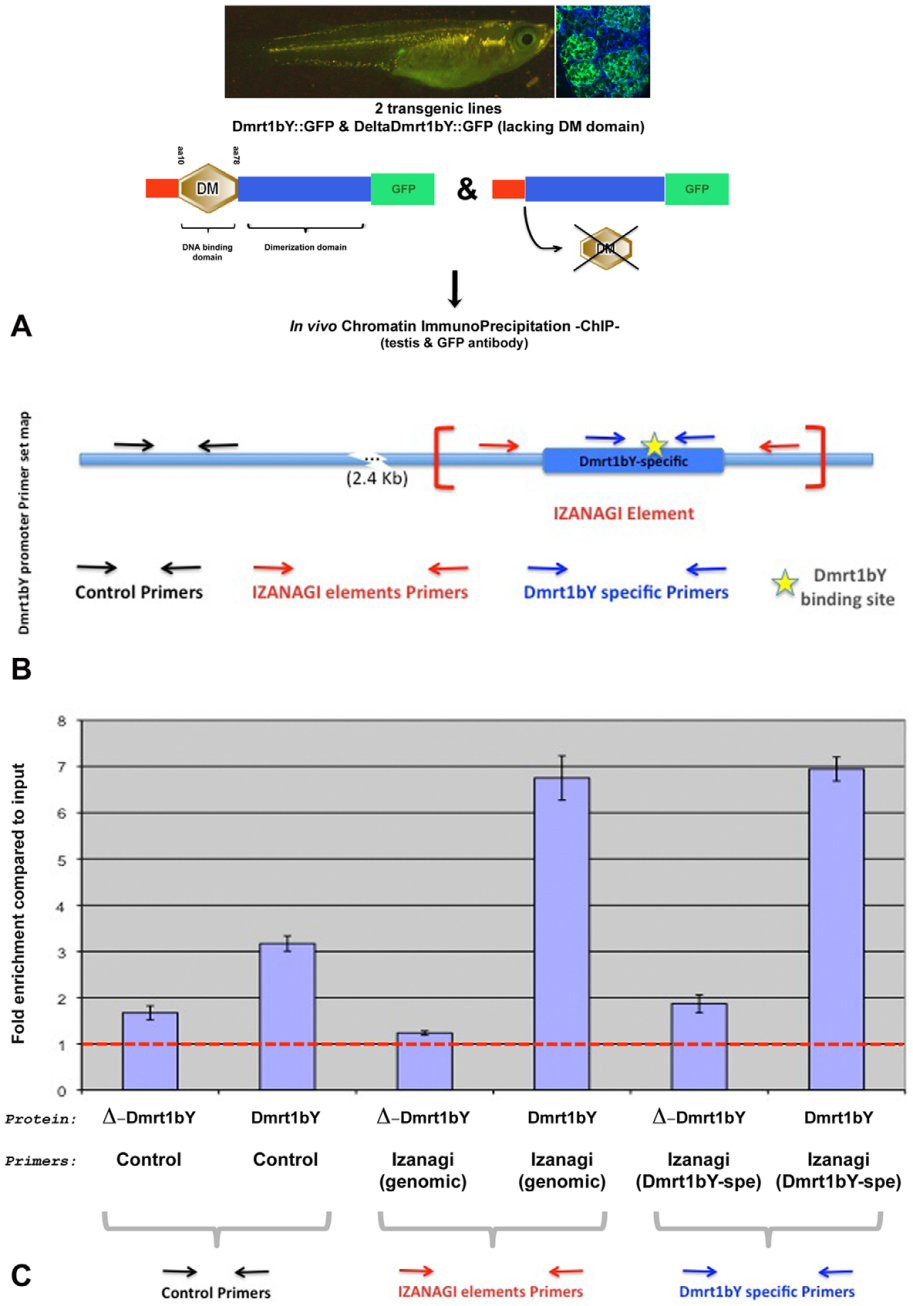
Tissue Chromatin immunoprecipitation (Tissue–ChIP) of Dmrt1bY binding to the *Izanagi* -nested Dmrt1 target site. Chromatin immunoprecipitation using both Dmrt1bY::GFP and deltaDmrt1bY::GFP transgenic lines respectively expressing either Dmrt1bY or a control truncated Dmrt1bY (delta DM form lacking the DNA binding domain) fused to GFP revealed specific *in vivo* Dmrt1bY protein affinity to the *Izanagi*-nested *dmrt1* target site, including the one described within *dmrt1bY* promoter. (A) Transgenic lines established for *in vivo* tissue–ChIP. (B) *Dmrt1bY* promoter primer sets map. (C) Specific enrichment of *Izanagi*-nested Dmrt1 binding sites subsequent to *in vivo* Dmrt1bY immunoprecipitation.

To assess *in vivo* Dmrt1bY interaction with its own promoter, two additional stable transgenic lines expressing either the full Dmrt1bY protein or a truncated form lacking the DNA binding domain, both fused to GFP, were created. The two lines were used for *in vivo* Tissue Chromatin ImmunoPrecipitation (Tissue-ChIP) on testis tissue using GFP antibody for immunoprecipitation. An up to 7-fold enrichment compared to the control confirmed the capacity of Dmrt1bY to bind not only to the Dmrt1 promoter-nested *Izanagi*-target site but also to the *Izanagi* Dmrt1bY-target site in general (Figure 6).

#### Expression domains of the two dmrt1 paralogs indicating cross-regulation during male gonad development *in vivo*

**Figure 7:**
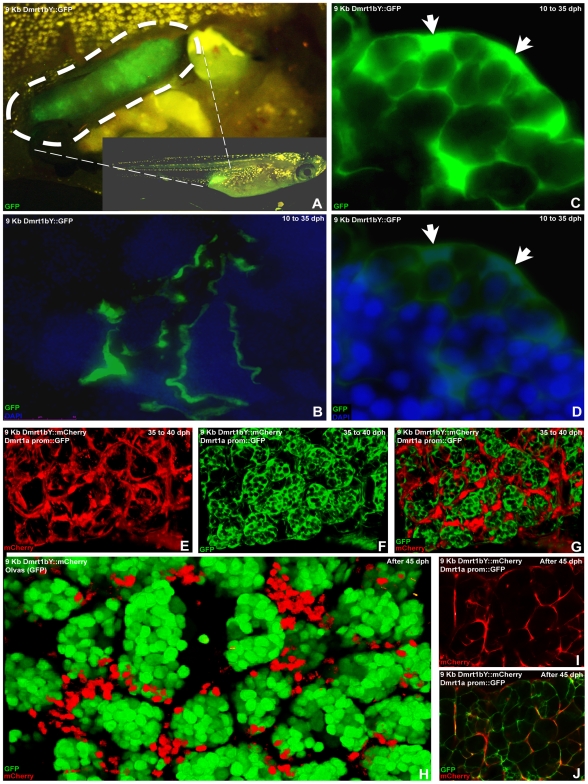
*In vivo* visualization of expression of the two medaka Dmrt1 paralogs. (A) Testis-specific GFP expression in 9KbDmrt1bYprom::GFP transgenic fish. (B) From testis formation (10 dph) up to adulthood, in 9KbDmrt1bYprom::GFP transgenic fish, robust testicular GFP expression is persistently noticeable in the epithelial cells of the intratesticular efferent duct. (C,D) Concomitantly, in agreement with the endogenous *dmrt1bY* expression, from 10 dph up to 30–35 dph strong specific GFP fluorescence is also detected in Sertoli cells. (E–G,I,J) By 35 dph, Sertoli cell-specific decline in fluorescence (mCherry) could be observed (E,I,H). In double transgenic fish (9KbDmrt1bYprom::mCherry and BACdmrt1a::GFP) this 9KbDmrt1bYprom-driven mCherry decline in expression is balanced by the rise of *dmrt1a* expression in Sertoli and germ cells (GFP in F,G,J). (H–J) In fully mature testes (after 45–50 dph), 9KbDmrt1bYprom-driven mCherry expression remains in scattered Sertoli cells around the germ cells (GFP in N) while Dmrt1a is now predominantly expressed in Sertoli cells (J).

Stable transgenic lines expressing fluorescent reporter protein (GFP and/or mCherry) were established to monitor the expression dynamics of Dmrt1a or Dmrt1bY *in vivo*. Analysis of early (10 to 30–35 dph) gonadal expression (Figure 7A) in two different (9KbDmrt1bYprom::GFP or 9KbDmrt1bYprom::mCherry) transgenic lines revealed identical fluorescent reporter protein expression in Sertoli cells (Figure 7C and 7D) as well as in interstitial tubule cells (Figure 7B). This exactly matches the protein expression pattern reported earlier from studies using a Dmrt1bY-specific antibody [42]. Later on, fluorescence declined in Sertoli cell (Figure 7E–7G to be compared to Figure 7H–7J). Noteworthy, corroborating the *in vitro* data, in double transgenic fish (9KbDmrt1bYprom::mCherry and BACdmrt1a::GFP) the decline of *dmrt1bY* promoter driven fluorescence was paralleled by a rise of *dmrt1a* promoter expression in Sertoli cells (Figure 7E–7G to be compared to Figure 7I and 7J). In fully mature testes (over 45–50 dph), *dmrt1bY* promoter expression remained only in few Sertoli cells scattered around the germ cells (Figure 7H) while the *dmrt1a* promoter was now predominantly expressed (Figure 7I and 7J).

## Discussion

Sex determination involves a complex hierarchy of genes. Expression screen analyses have resulted in hundreds of candidate genes that show sex-specific expression pattern. However it has been difficult to place these genes into a network of gene regulation and function. Nevertheless, several genes encoding for transcription factors, with specific temporal and spatial expression patterns during early gonad induction, have been suggested to participate in this process. Among them, from *C. elegans* to mammals, genetic evidence has suggested that the *dmrt1* gene is an important regulator of male development at a downstream position of the regulatory network. In medaka, a duplicated copy of *dmrt1* has acquired the upstream position of the sex-determining cascade. The analysis presented here provides evidences that this evolutionary novelty, which is predicted to require a rewiring of the regulatory network is brought about by co-option of “ready-to -use” pre-existing *cis*-regulatory elements carried by transposing elements. We could show that the master sex determining gene of medaka, *dmrt1bY*, is able to bind to one of these elements in its own promoter. This binding leads to a significant repression of its own transcription.

**Figure 8:**
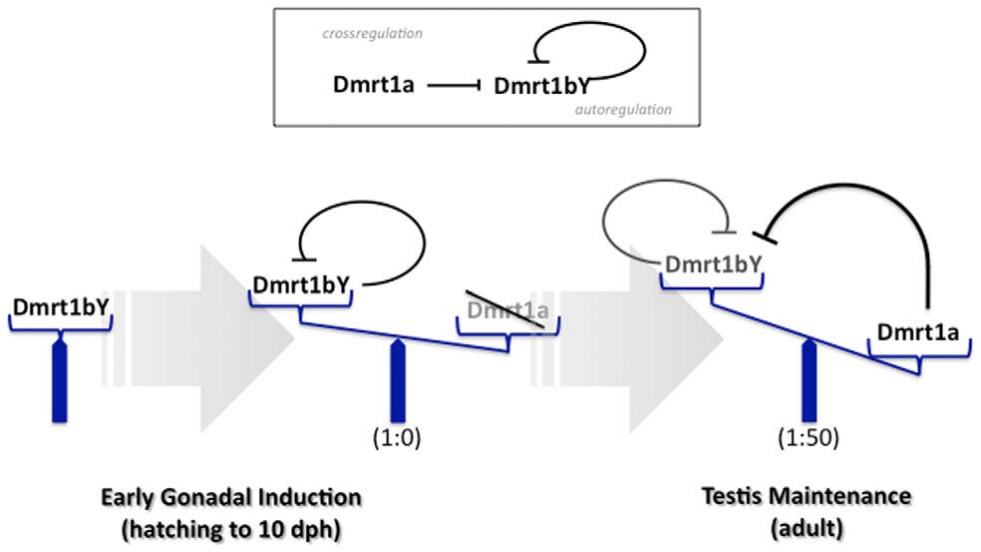
Model for feedback and cross-regulation of the medaka dmrt1 paralogs. During sex determination stages only *dmrt1bY* is expressed and *dmrt1a* is off. Hence, the sex determining function of *dmrt1bY* is exerted. In adult testes, both paralogs are expressed notably in Sertoli cells, but the autorepression of the *dmrt1bY* promoter by its own gene product and the cross-regulation by Dmrt1a lead to a predominant expression of *dmrt1a* compared to *dmrt1bY* (appox. 50 fold).

During early stages when the primordial gonad is formed, *dmrt1bY* is exclusively expressed and exerts its sex determining function [18]. The *dmrt1a* gene, with its proposed specification and maintenance function for the Sertoli cells, is expressed only when the testes are in the process of differentiation. Notably, the master sex determinator gene *dmrt1bY*, continues to be expressed. In adult testes, where both paralogs have been shown to be expressed, the predominant expression of *dmrt1a* compared to *dmrt1bY* (50 fold higher; [43]) argues for a downregulation of *dmrt1bY*. Although additional post-transcriptional mechanisms accounting for *dmrt1bY* expression regulation, involving the 3′ UTR [44], have been shown to be essential for spatial expression pattern in the embryo and restricted expression to the gonad in adult fish, the data presented here indicate that a feed back auto-regulation of *dmrt1bY* promoter activity and trans-regulation by its paralog Dmrt1a is a key mechanism of *dmrt1bY* transcriptional tuning (Figure 8).

With respect to the evolutionary history of the two *dmrt1* genes in medaka, it is of note that the newly generated paralog *dmrt1bY*, independently of any functional considerations, is kept back under tight transcriptional regulation of the ancestral *dmrt1a* gene. Consequently this avoids any kind of expression pattern redundancy in testes after their development is initiated and could then be a reasonable way of preserving both genes from any purification/degeneration processes after duplication, thus favouring a subsequent sub-neo-functionalization.

So far no putative Dmrt1 binding site could be observed within the more than 10 Kb upstream medaka *dmrt1a* sequence inspected. Similarly such Dmrt1 target sites are absent from the zebrafish, fugu, stickleback, mouse or human 10 kB upstream *dmrt1* promoter regions. This together with the apparent loss of Dmrt1 canonical *cis*-regulatory sequences (such as Gata4) indicates a particular transcriptional context acquired by *dmrt1bY* during its evolution towards becoming a novel master sex determination gene.

It was previously reported that multiple TEs inserted into the Y-specific region on medaka LG1 [30]. Interestingly, our study revealed that the *cis*-regulatory element containing the Dmrt1 binding site, pre-existing within the *Izanagi* element at the time of its insertion, was co-opted in order to confer *dmrt1bY* its specific expression pattern after gene duplication around 10 million years ago [19],[41]. This fact has interesting evolutionary implications, since TEs are probably the most dynamic part of the genome. Dmrt1 possibly also regulates other genes in the proximity of *Izanagi* elements via the Dmrt1 binding site (Table S1).

In the context of gene duplication and its correlated process of sub-/neo- functionalization (see [45]–[47] for review), the contribution of TEs to the remodelling of the sex determination cascade (see [12],[48] for review) is of prime interest. The case reported here for the medaka-specific *Izanagi* element bringing in a novel regulatory element into the *dmrt1bY* promoter is –at least to our knowledge- the first example showing that TEs not only change/rewire the expression of existing genes but surely lead to the creation of new regulatory hierarchies within recently duplicated genes. The present case is even more interesting since this new TE-derived TFBS confers transcriptional control from the ancestral gene against the duplicate and allows the *dmrt1bY* gene to take an upstream position in the sex determination cascade without excluding its *dmrt1* ancestor from a role in sexual development.

This supports a role of TEs for transcriptional network rewiring in sub- and/or neo- functionalization of duplicated genes in creating new hierarchies of sex determining genes.

## Materials and Methods

### Bioinformatic analyses

Comparative analysis of vertebrate *Dmrt1* genomic regions were performed with mVISTA at http://genome.lbl.gov/vista [49] using the Shuffle-LAGAN alignment program [50]. Medaka *dmrt1a* (LG9) and *dmrt1bY* (LG1) region sequences were obtained from [30], all other regions from the Ensembl Genome Browser (http://www.ensembl.org/; release 49, March 2008): stickleback groupXIII, scaf57; Fugu scaf4; Tetraodon chr12, scaf14966; zebrafish chr5, scaf463; chicken chrZ, supercontig194; human chr9, supercontig NT_008413.

Screens for repetitive elements were performed with RepeatMasker (http://www.repeatmasker.org/). Additional copies of repeat elements and their genomic environment in the medaka genome (version HdrR, Oct 2005) were identified with BLASTN with >85% sequence identity over >85% of query length (Table 1). Alignments of repeat elements were obtained with CLUSTALW as implemented in BioEdit [51] followed by manual improvement. 50% threshold frequency was used for inclusion in repeat consensus sequences. The putative THAP domain found in the *Izanagi* consensus was identified by comparison to the PFAM database (http://pfam.sanger.ac.uk/).

Transcription factor binding sites were determined using MatInspector of the Genomatix portal (http://www.genomatix.de/). Binding sites for Dmrt1 in different genomes were identified using the matrix provided by [40] together with the Regulatory Sequence Analysis Tools portal; RSat (http://rsat.ulb.ac.be/rsat/). MEGA4 [52] was used to estimate sequence divergence between repeat 1 and the *Izanagi* element consensus (0.034 +− 0.005) as well as between repeat 2 and the *Rex1* element consensus (0.010 +− 0.003) using the Kimura-2-parameter model. Linearized neighbor-joining trees of *dmrt1* and *tyrosinase a* gene were obtained as described in ref. [41], with the only exception that they were based on third codon positions only. Other models of sequence evolution gave similar results. Accession numbers are given in Figure S6.

### Cloning of the 9.107 Kb 5′ flanking sequence of medaka Dmrt1bY and plasmid constructs

For promoter analysis, a 9107 bp fragment upstream of the Dmrt1bY open reading frame (ORF) was isolated by restriction enzyme digestion (*XhoI/EcoRI*) from BAC clone Mn0113N21 [30], was cloned into pBSII-ISceI plasmid (pBSII-ISceI::9 Kb Dmrt1bY prom. plasmid). Subsequently, *Gaussia* luciferase gene from pGLuc-basic (New England Biolabs) plasmid was inserted between *EcoRI* and *NotI* sites of pBSII-ISceI::9 Kb Dmrt1bY prom (pBSII-ISceI::9 Kb Dmrt1bY prom::GLuc plasmid). pBSII-ISceI::3 Kb Dmrt1bY prom::GLuc and pBSII-ISceI::6 Kb Dmrt1bY prom::GLuc plasmids were constructed the same way removing 5′ fragments of the 9107 bp Dmrt1bY promoter region using *Eco47III* and *HindIII* restriction enzyme digestion respectively and re-ligation.

Mutation of the Dmrt1bY binding site was performed by PCR in the context of pBSII-ISceI::3 Kb Dmrt1bY prom::GLuc plasmid (native form: AATGCATTGTTGCAG; mutated form: GCCGGCTTCCCACCA). All PCR-obtained fragments were sequenced.

To generate plasmids for *in vitro* transcription, full-length cDNAs encoding medaka *dmrt1a* or *dmrt1bY* were subcloned into *EcoRI/NotI* digested pRN3 plasmid [53].

For establishment of transgenic lines, either GFP or mCherry open reading frames were inserted between *EcoRI* and *NotI* sites of pBSII-ISceI::9 Kb Dmrt1bY prom (pBSII-ISceI::9 Kb Dmrt1bY prom::GFP or mCherry plasmids respectively). GFP fusion protein vector (dmrt1bY::GFP and deltadmrt1bY::GFP) were constructed as described in [43].

### Cell lines, cell transfection, and Luciferase assay

Mouse TM4 Sertoli cells, *Xiphophorus* embryonic epithelial A2 cells, and medaka spermatogonial (Sg3) and fibroblast like (HN2) cells were cultured as described [54],[55],[56],[57]. Cells were grown to 80% confluency in 6-well plates and transfected with 5 µg expression vector using FuGene (Roche) or Lipofectamine (Invitrogen) reagents as described by the manufacturers.

*Gaussia* luciferase activity was quantified using the Luciferase Reporter Assay System from Promega and normalized against co-tranfected firefly luciferase expressing plasmid (ptkLUC+; [58]). When DNA amounts transfected are expressed as a ratio, the total amount of expression vector remained constant (5 µg) by filling in the reaction with empty vector. Experiments for which error bars are shown result from at least three replicates and error bars represent the standard error of the mean.

### Electrophoretic Mobility Shift Assays (EMSA)

(Dmrt1bY-Trgt) 5′-AGCTTAATGCATTGTTGCAGAGCT-3′, (Competitor) 5′-AGCTGACGGCCGCGAAGCAAGCT and respective complements were annealed by heating to 90°C for five minutes in 1X T4 PolyNucleotide Kinase (PNK) buffer (70 mM Tris-HCl (pH 7.6), 10 mM MgCl_2_, 5 mM dithiothreitol); slow-cooled to 50°C; held at that temperature for 5 minutes and then cooled to room temperature. For radioactive labelling 50 pmol of the duplex 5′ termini were used together with 50 pmol of gamma-[^32^P]-ATP and 20 units of T4 PNK in 1X adjusted T4 PNK buffer and incubated for 20 minutes at 37°C. Unincorporated nucleotides were removed through a Sephadex G-50 spin column.

For producing Dmrt1a and Dmrt1bY proteins, pRN3::Dmrt1a or pRN3::Dmrt1bY plasmids were linearized using *KpnI* and then *in vitro* transcribed using mMessage mMachine kit (Ambion). Finally, Dmrt1a or Dmrt1bY proteins were *in vitro* translated using Ambion's Retic Lysate Kit from the previously *in vitro* transcribed capped *Dmrt1a* or *Dmrt1bY* RNAs.

DNA binding reaction contained 10 mMTris-HCl (pH 7.9), 100 mM KCl, 10% glycerol, 5 mM MgCl_2_, 1 µg torula rRNA, 0.075% Triton X-100, 1 mM DTT, 1 µg BSA, 0.5 ng radiolabeled duplex probe and 2 or 4 µL *in vitro* translation mix in a total volume of 20 µL. 1/10 volume heparin (50 mg/mL) was added just before loading the binding reaction. For control reticulocyte lysate alone together with radiolabeled duplex probe was used and did not result in any shift (data not shown). Binding reactions were performed on ice for ten minutes and complexes were resolved on a 5% native acrylamide (37.5∶1) gel in 0.5 X TBE and then directly subjected to autoradiography.

### Expression analyses

Total RNA was extracted from 9KbDmrt1bYprom::Dmrt1bY::GFP::Dmrt1bY3′UTR transgenic fish (Carbio genetic background) using the TRIZOL reagent (Invitrogen) according to the supplier's recommendation. After DNase treatment, reverse transcription was done with 2 micrograms total RNA using RevertAid First Strand Synthesis kit (Fermentas) and random primers. Real-time quantitative PCR was carried out with SYBR Green reagents and amplifications were detected with an i-Cycler (Biorad). All results are averages of at least two independent RT reactions and 2–5 PCR reactions from each RT reaction using each time three set of primer combination (DMTYk: 5′-CCTTCTTCCCCAGCAGCCT-3′/eGFP3: 5′-AGTCGTGCTGCTTCATGTGGTC-3′; DMTYa2: 5′-CGACTCCATGAGCAGTGAAA-3′/eGFP3: 5′-AGTCGTGCTGCTTCATGTGGTC-3′; DMTYa2: 5′-CGACTCCATGAGCAGTGAAA-3′/eGFP5: 5′-GAACTTCAGGGTCAGCTTGC-3′. Error bars represent the standard deviation of the mean. Relative expression levels (according to the equation 2–DeltaCT) were calculated after correction of expression of elongation factor 1 alpha *(elf1alpha)* and brain expression was set to 1 as a reference.

### *In vivo* Chromatin Immunoprecipitation

For *in vivo* chromatin immunoprecipitation, the EpiQuik Tissue Chromatin Immunoprecipitation kit (Epigentek) was utilized according to the manufacturers instructions, using testis tissue samples either from dmrt1bY::GFP or deltadmrt1bY::GFP transgenic fish (20 testes for each) and GFP antibody (Upstate) for immunoprecipitation. After immunoprecipitation [(*Izanagi* element Dmrt1bYspeF003) 5′-TCCGGTCTCTCCGGCGTGTGG-3′/(*Izanagi* element *Dmrt1bYspeR00*) 5′-TTGTAAGAGGACCTGCAACAATG-3′; (*Izanagi* element F01) 5′-CTATCTTGGTGAGGTCGACGATGCC-3′/(*Izanagi* element R01) 5′-AATTTAAATTACATGTCAAAGAGGTC-3′; (Dmrt1bYCtrF04) 5′-GTTCTGACTTTCAGCGTCTCACCTG-3′/(Dmrt1bYCtrR04) 5′-GGTTCTGGTCCAAATCTGTCAGAAG-3′] primer sets were used for enrichment quantification by real-time PCR.

### Transgenic fish lines

For the generation of stable transgenic lines the meganuclease protocol [59] was used. Briefly, approximately 15–20 pg of total vector DNA in a volume of 500 pl injection solution containing I-*Sce*I meganuclease was injected into the cytoplasm of one cell stage medaka embryos (Carbio strain). Adult F0 fish were mated to each other and the offspring was tested for the presence of the transgene by PCR from pooled hatchlings. Siblings from positive F1 fish were raised to adulthood and tested by PCR from dorsal fin clips as described [60].

Identically to the transgenic line expressing the Dmrt1bY protein fused to GFP [61] a second line lacking the Dmrt1bY DNA binding domain (DM-domain between aminoacids 10 and 78) was established. These two lines were used for *in vivo* Chromatin Immunoprecipitation. Similarly, for *in vivo* Dmrt1bY promoter activity quantification another transgenic line expressing a 9Kbdmrt1bYprom driven dmrt1bY::GFP fusion protein was created. Dmrt1a prom::GFP transgenic medaka was generated following the BAC transgenic method [62]. The BAC clone including dmrt1a genomic region, ola1-171C06 (NCBI accession numbers; DE071574 and DE071575) was obtained from NBRP. The followings were the primers to amplify EGFP fragments for homologous recombination into the BAC clone; Forward: 5′ -tctgacatgagcaaggagaagcagggcaggccggttccggagggcccggcTCAACCGGTCGCCACCATGG-3′ Reverse:5′-ttcagcggagacacgaagccgtggttccggcagcgggagcacttgggcatcGTCGACCAGTTGGTGATTTTG-3′.

## Supporting Information

Figure S1 mVISTA plots of vertebrate *Dmrt1* upstream regions. Medaka Y chromosomal *dmrt1bY* region (upper part) and autosomal *dmrt1a* region (LG9; lower part) are used as references. Regions I–IV contribute to length differences between the medaka *dmrt1* upstream regions. Dark blue and green indicate exons of genes and pseudogenes, respectively, light blue and green untranslated regions. Red indicates conserved non-coding sequences. Conservation of medaka *dmrt1* promoters with other vertebrates is restricted to the *MHCL* pseudogene regions.
(3.97 MB PDF)

Figure S2 Annotation of the *dmrt1bY* promoter. *KIAA0172p* and *MHCLbp* regions are green shaded, regions IV–III and repeat 2 are grey shaded. The *Izanagi* element (repeat 1) is red shaded, its terminal inverted repeats are black shaded and the 8 bp target site duplication is underlined. The three identified putative THAP domain-encoding exons are pink shaded. The dmrt1bY exon 0 is blue shaded. Red lines mark border segments for the transcriptional regulation analysis (“3 Kb”, “6 Kb”, “9 Kb” promoter). See also Figure 1 and Figure S4.
(0.02 MB PDF)

Figure S3 Putative THAP domain in the *Izanagi* element. (A) Structure of the *Izanagi* element. Pink boxes indicate the three putative exons constituting the THAP domain are. The insertion of repeat 2 in the *dmrt1bY* promoter splitting repeat 1 into repeat 1b is indicated by the arrow. Note that the *Izanagi* element is shown here in reverse complement compared to the *dmrt1bY* promoter. (B) Alignment of the putative THAP protein domain from the *Izanagi* element consensus sequence with the THAP domain from the PFAM database. Identical essential residues are yellow shaded; other identical residues are blue shaded. + indicates similar residues.
(1.01 MB PDF)

Figure S4 Repeat elements and transcription factor binding sites in the *dmrt1bY* promoter. The three segments correspond to the regions used for transcriptional regulation analysis. Transcription factor binding sites conserved with the *dmrt1a* promoter are boxed. Of particular importance for transcriptional regulation of *dmrt1bY* might be the repeat 1b area (beige box) with multiple Sox5 and Pax2 binding sites, as well as a Dmrt1 binding site (red). Further upstream, two SF1 binding sites are located (red). For further characterization see Figure 1A and Table 1. Prog Rec: progesterone receptor; Est Rec: estrogen receptor; And Rec: androgen receptor binding sites.
(2.37 MB PDF)

Figure S5 Activity of *dmrt1bY* promoter deletion constructs in different cell lines. (A–C) Various 5′-deletions mutants (3 Kb and 6 Kb) from pBSII-ISceI::9 Kb *dmrt1bY* prom::GLuc plasmid were transfected either into Mouse TM4 Sertoli, *Xiphophorus* embryonic epithelial A2 or medaka HN2 fibroblast like cells. Transfections were repeated three times; error bars represent the standard errors of the means.
(0.62 MB PDF)

Figure S6 Timing of repeat insertions into the *dmrt1bY* promoter. Linearized NJ trees for *dmrt1* (A) and *tyra* (B) genes based on third codon positions using the Kimura-2-parameter model are shown. The split between fugu and *Oryzias* species was set to 95 million years ago (MYA) [41]. The sequence divergence between repeats 1 and 2 and their consensus sequence, respectively, is indicated. The repeat 1 origin falls onto the branch, at which the *dmrt1* gene duplication has occurred [41] and is younger than the inferred *dmrt1* duplication period (blue). The repeat 2 insertion is very recent. Other models of sequence evolution gave similar results.
(2.49 MB PDF)

Figure S7 Transient transfection analysis of the Dmrt1bY promoter nested Dmrt1 binding site. Transcriptional activity of the mutant 3 Kb proximal Dmrt1bY promoter (mutated Dmrt1 binding site) was not significantly impaired while overexpressing dmrt1bY or not.
(1.78 MB PDF)

Table S1 Location and adjacent genes of repeat 1 elements containing Dmrt1 binding sites in the medaka genome.
(0.06 MB DOC)

Text S1 Izanagi and Izanami: Creators of Japan.
(6.88 MB PDF)
