# Supplementary figures and images for: Transcriptional Rewiring of the Sex Determining dmrt1 Gene Duplicate by Transposable Elements
Source: PLoS Genet. 2010 Feb 12;6(2):e1000844. doi: 10.1371/journal.pgen.1000844 (PMC2820524; doi:10.1371/journal.pgen.1000844)

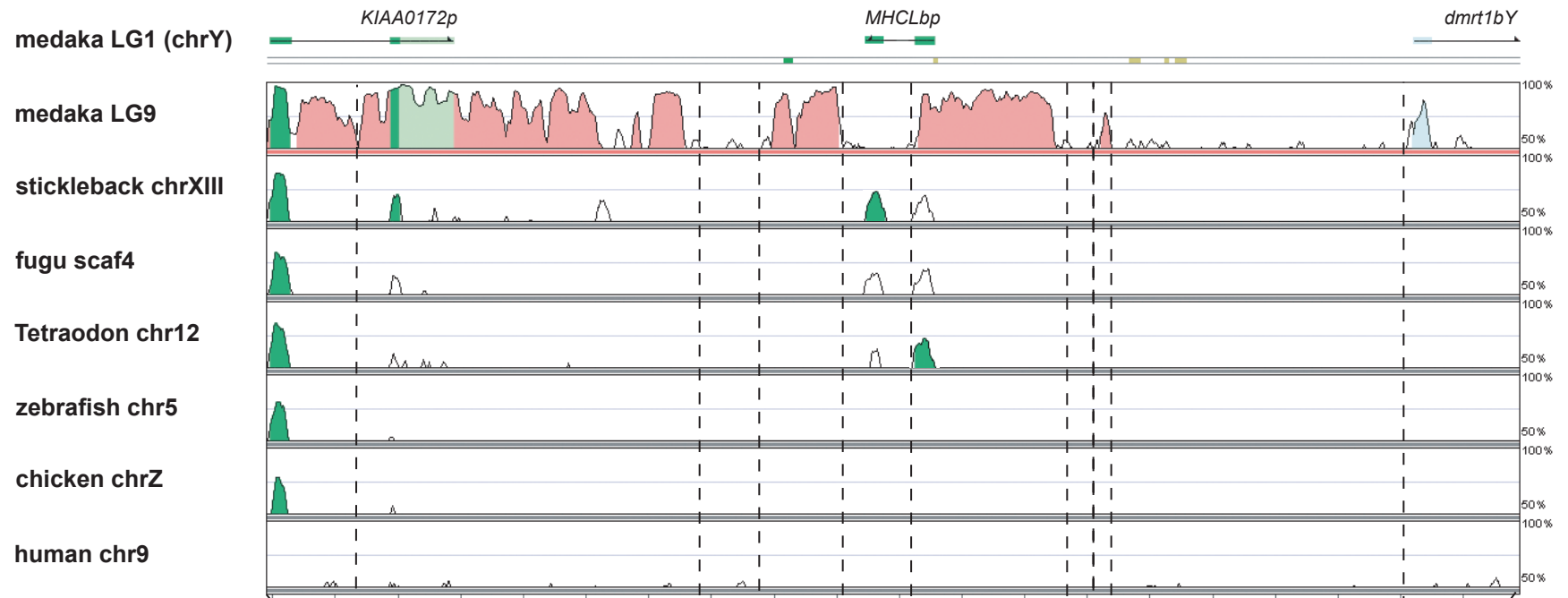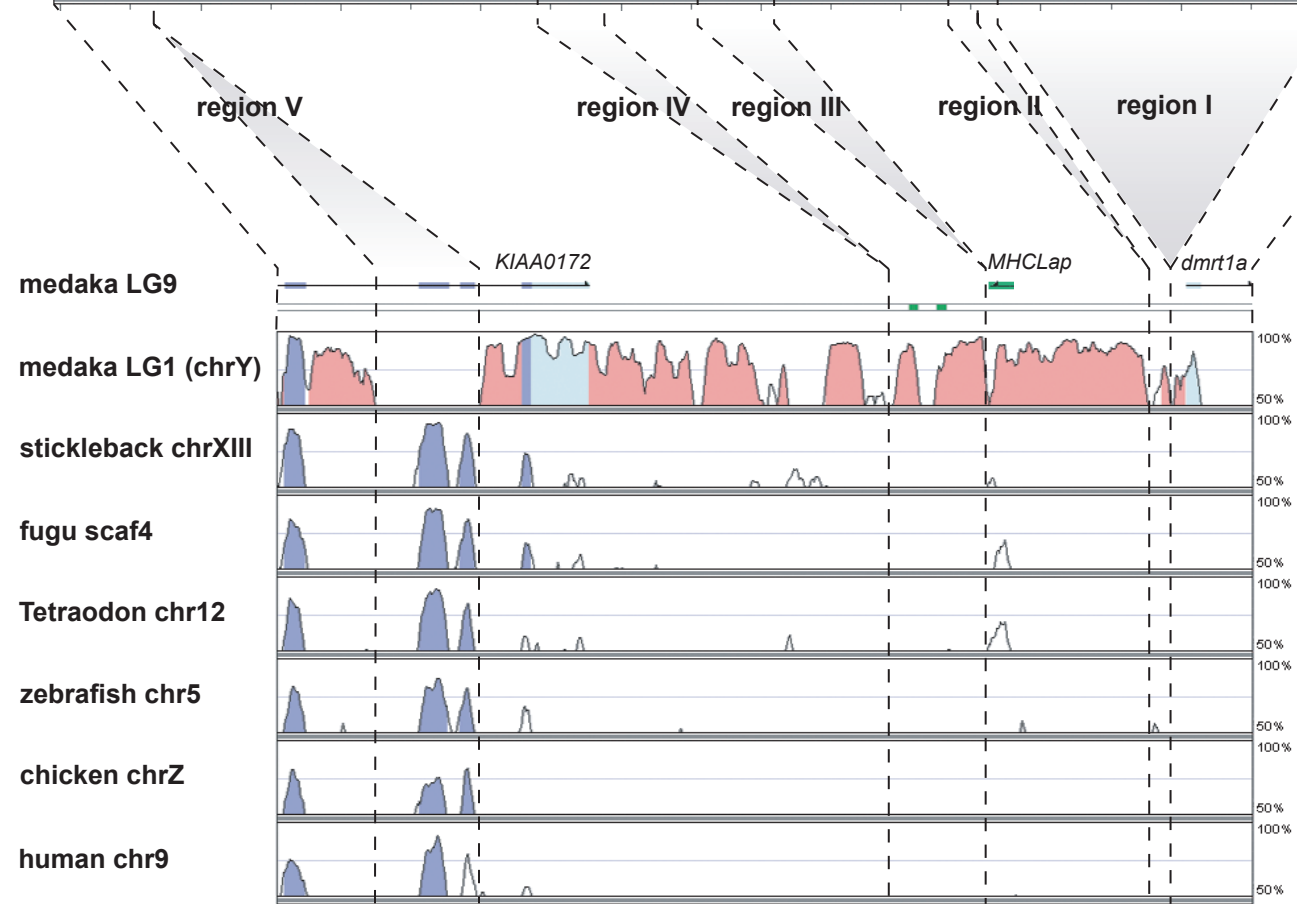

Supplement: Figure S1 — mVISTA plots of vertebrate Dmrt1 upstream regions. Medaka Y chromosomal dmrt1bY region (upper part) and autosomal dmrt1a region (LG9; lower part) are used as references. Regions I–IV contribute to length differences between the medaka dmrt1 upstream regions. Dark blue and green indicate exons of genes and pseudogenes, respectively, light blue and green untranslated regions. Red indicates conserved non-coding sequences. Conservation of medaka dmrt1 promoters with other vertebrates is restricted to the MHCL pseudogene regions. (3.97 MB PDF) [file pgen.1000844.s001.pdf]

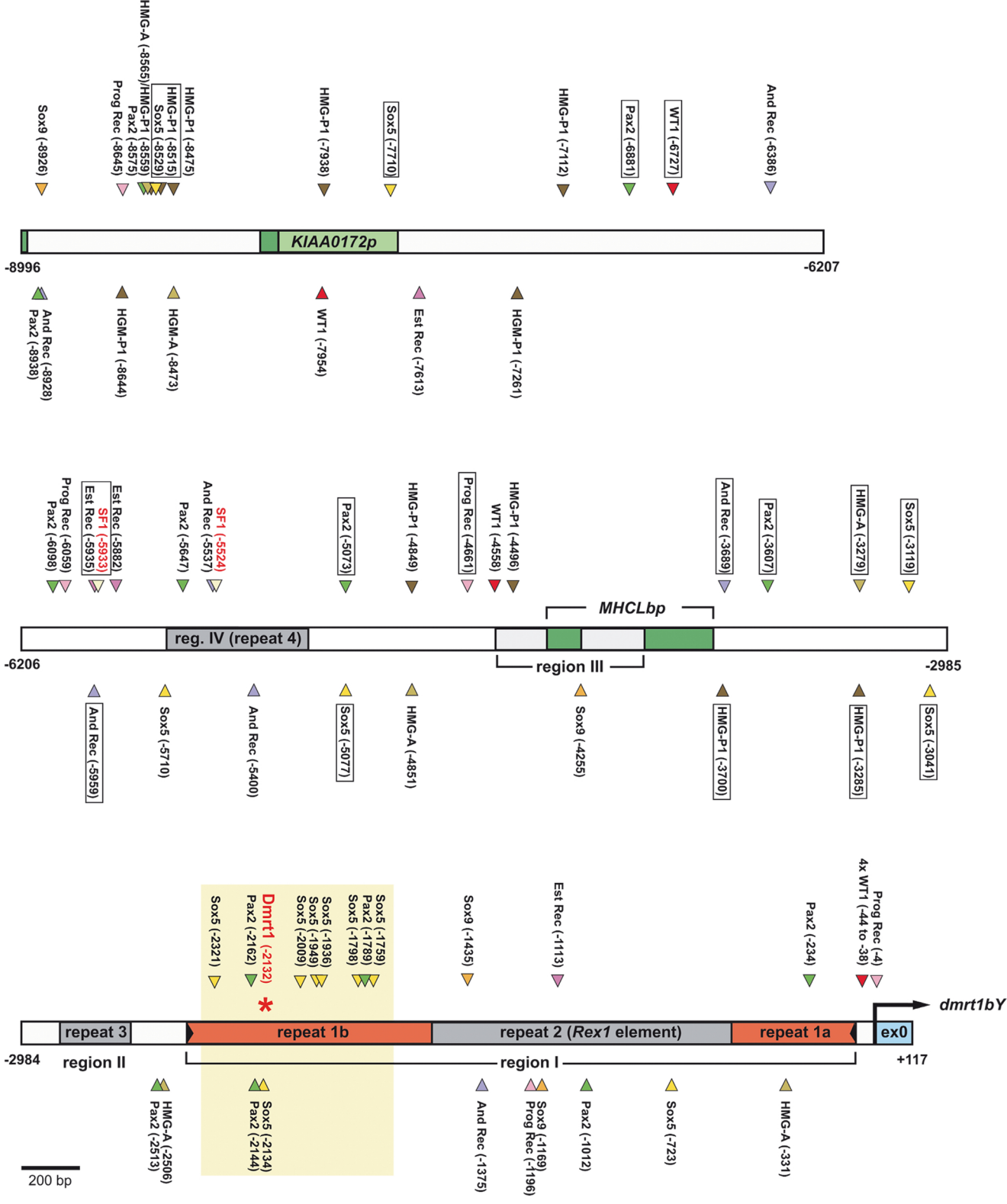

Supplement: Figure S4 — Repeat elements and transcription factor binding sites in the dmrt1bY promoter. The three segments correspond to the regions used for transcriptional regulation analysis. Transcription factor binding sites conserved with the dmrt1a promoter are boxed. Of particular importance for transcriptional regulation of dmrt1bY might be the repeat 1b area (beige box) with multiple Sox5 and Pax2 binding sites, as well as a Dmrt1 binding site (red). Further upstream, two SF1 binding sites are located (red). For further characterization see Figure 1A and Table 1. Prog Rec: progesterone receptor; Est Rec: estrogen receptor; And Rec: androgen receptor binding sites. (2.37 MB PDF) [file pgen.1000844.s004.pdf]

A) *dmrt1*

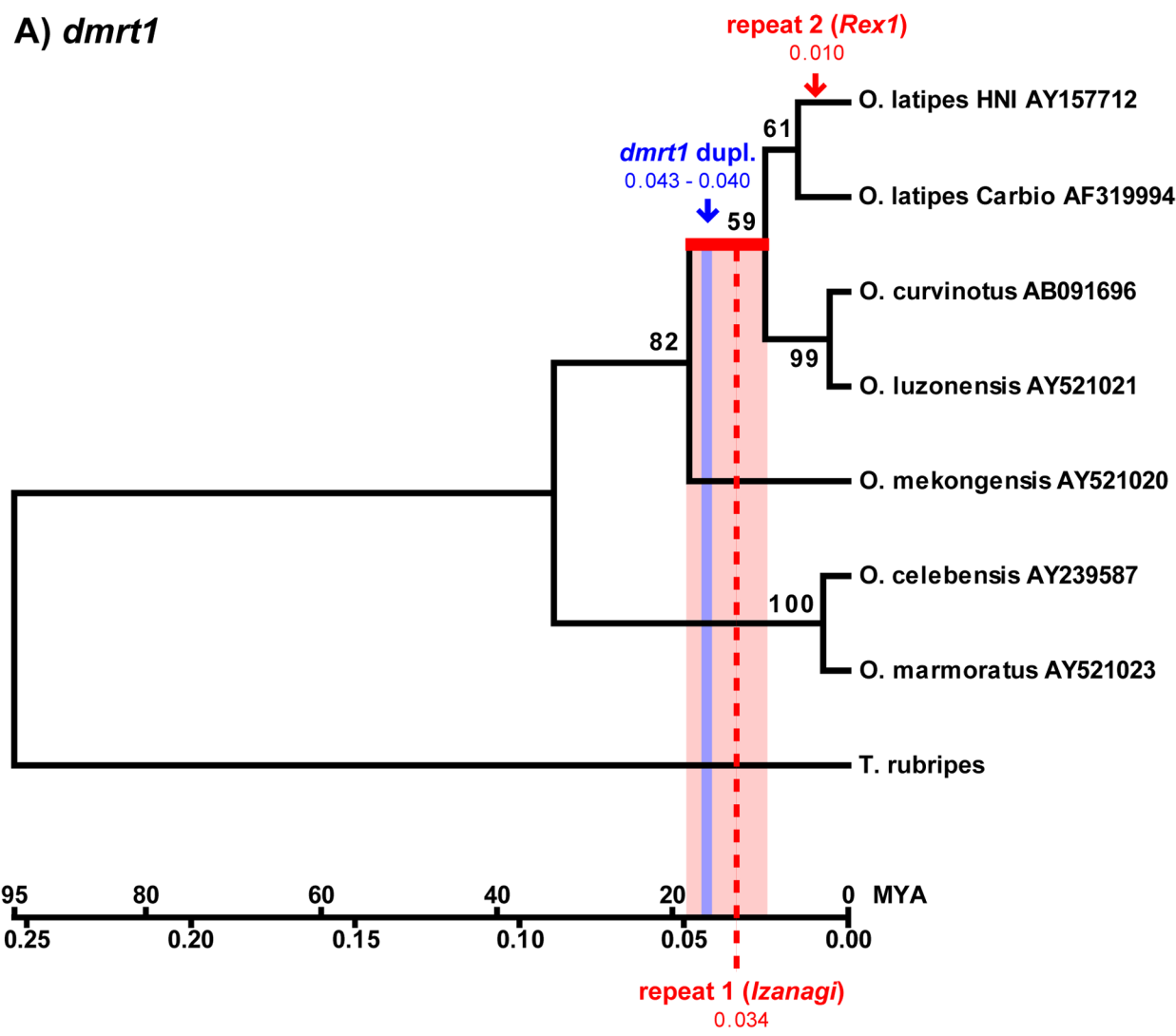

B) *tyra*

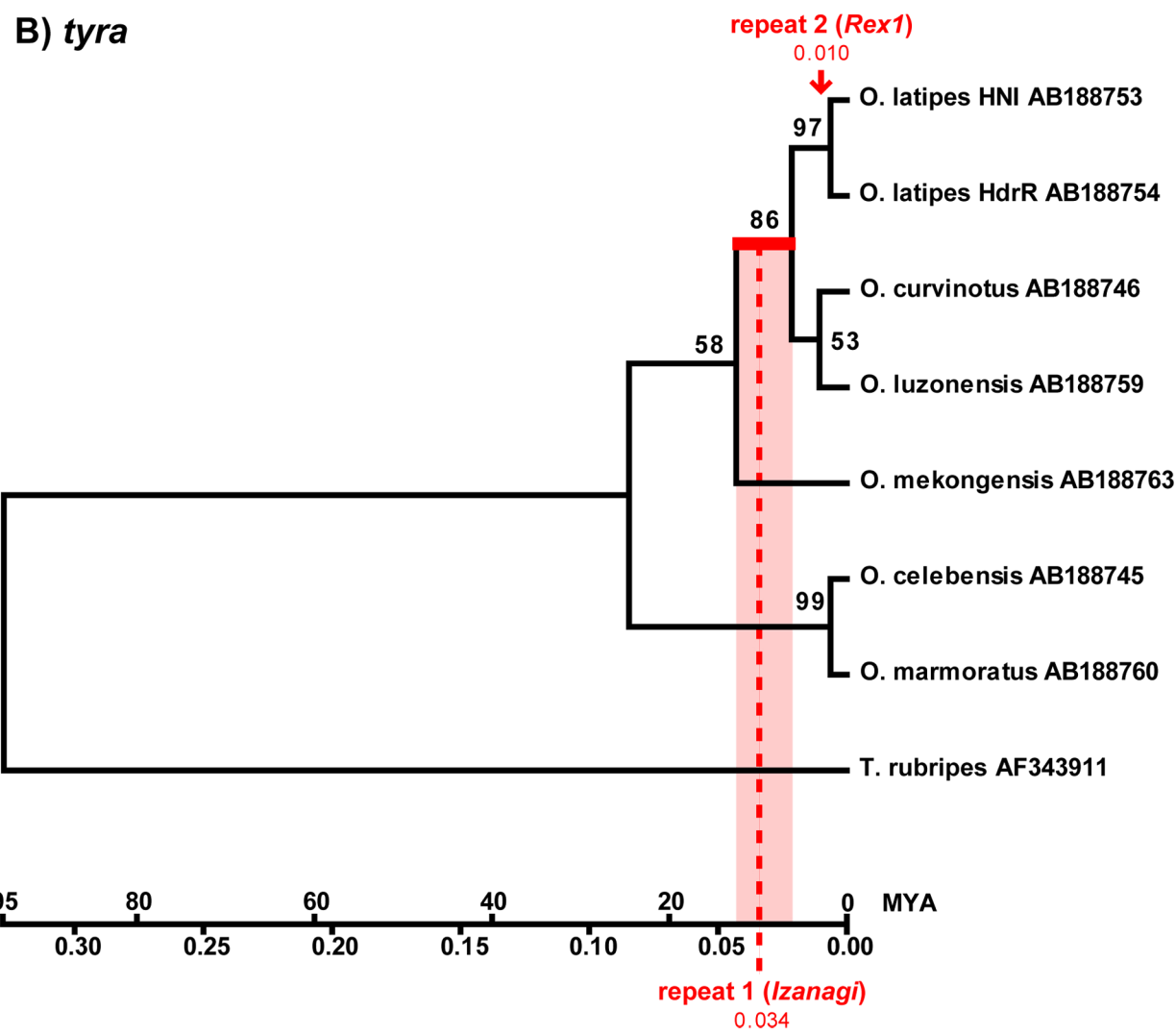

Supplement: Figure S6 — Timing of repeat insertions into the dmrt1bY promoter. Linearized NJ trees for dmrt1 (A) and tyra (B) genes based on third codon positions using the Kimura-2-parameter model are shown. The split between fugu and Oryzias species was set to 95 million years ago (MYA) [41]. The sequence divergence between repeats 1 and 2 and their consensus sequence, respectively, is indicated. The repeat 1 origin falls onto the branch, at which the dmrt1 gene duplication has occurred [41] and is younger than the inferred dmrt1 duplication period (blue). The repeat 2 insertion is very recent. Other models of sequence evolution gave similar results. (2.49 MB PDF) [file pgen.1000844.s006.pdf]

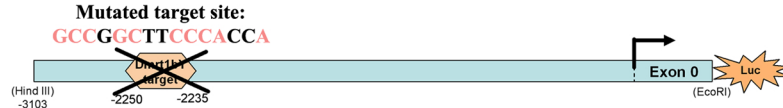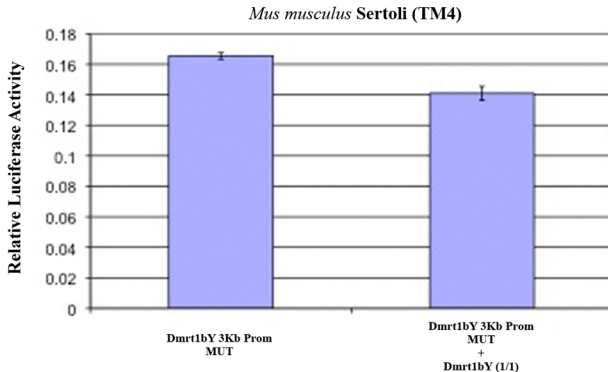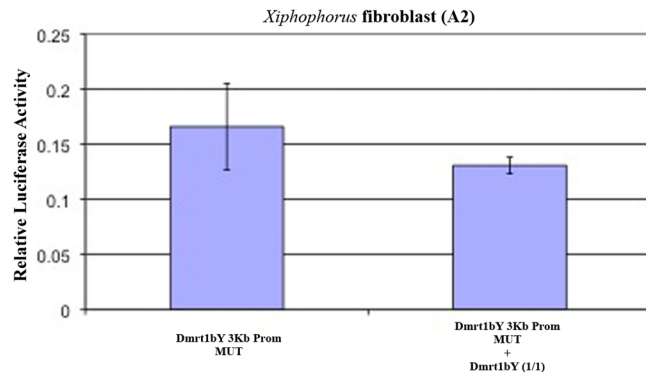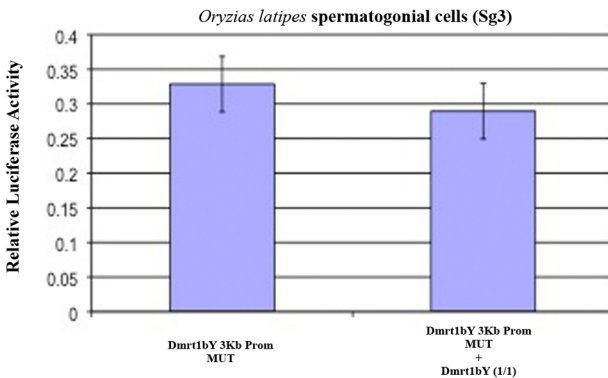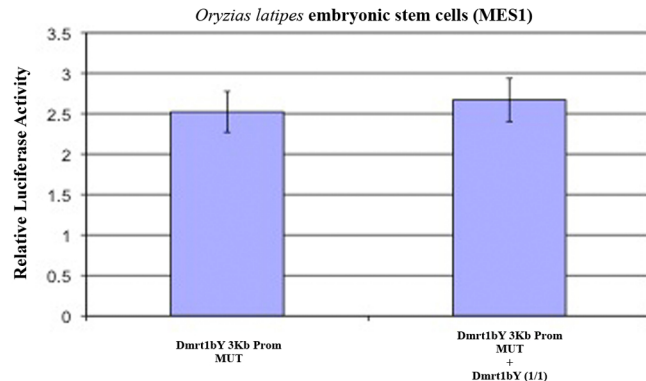

Supplement: Figure S7 — Transient transfection analysis of the Dmrt1bY promoter nested Dmrt1 binding site. Transcriptional activity of the mutant 3 Kb proximal Dmrt1bY promoter (mutated Dmrt1 binding site) was not significantly impaired while overexpressing dmrt1bY or not. (1.78 MB PDF) [file pgen.1000844.s007.pdf]
