# Supplementary material for: Transcriptional Rewiring of the Sex Determining dmrt1 Gene Duplicate by Transposable Elements
Source: PLoS Genet. 2010 Feb 12;6(2):e1000844. doi: 10.1371/journal.pgen.1000844 (PMC2820524; doi:10.1371/journal.pgen.1000844)
Supplement: Figure S2 — Annotation of the dmrt1bY promoter. KIAA0172p and MHCLbp regions are green shaded, regions IV–III and repeat 2 are grey shaded. The Izanagi element (repeat 1) is red shaded, its terminal inverted repeats are black shaded and the 8 bp target site duplication is underlined. The three identified putative THAP domain-encoding exons are pink shaded. The dmrt1bY exon 0 is blue shaded. Red lines mark border segments for the transcriptional regulation analysis (“3 Kb”, “6 Kb”, “9 Kb” promoter). See also Figure 1 and Figure S4. (0.02 MB PDF) [file pgen.1000844.s002.pdf]

>dmrt1bY promoter

CTCGAGCCAGCCAGGTGAGACGTTGAAGCTCTCCACCAATCAGGTAGCAGCTTAACCACGTTTCACAGCAGAACACAAAG
CACGTTGACATTTAAGCAACACCTGGTGGTCAGGCAATCCTTGTGAACTGCTGTGGTTGAGGTGTGGGACGGGTACCGTT
TGAATTTCAGCCGGTGCTAAACCGGTCCTTTGGAAACGGTTTTGGCAGCTGAATGCGCCCGCATCGGTGAAAACGCTTGA
CTTTTTCCTGTAAACATGAAAAGTGCACCAAAGCAGCATGAGGGACAGCCTTTTTTCATCTATGTGGTGCTCGGATCCAT
CAGAACCGTCTGATGTGAAGTTCGGCAGAACCTTTCATTCAGTGTTCTCACTGAAACTCCGCCCATCAATAAGGCCAACC
CATTCCTATATGAGCCCGCCTACAATCTCCAGGAATTAAAAATGACTGAAGCAAAACTGCACAGGAAACAAACAATGGAC
GCGTCGGGAATGAAGCACGATCGTTGTTTTTCTGTGGTCTGGTGGTACTGAATGCATCTGTAATGGTACCACTGCTGGGT
TCTGGACACTGAGATGATGAAGATGCTGAAGGTGATGAAGATGCTGAGGTTCTGGATGTGATCAGGAAGAGTCCATCAGT
TGATCAGATCATGGTGAATGTGCTGCAGATCAGAGCAGGGAAGCAGATGGAGGAACAGGGATGAGTATGCTGACAGGATT
CTGAGGAGAGACCACAGAGGAGGTTCATGGATGAGGGAGGAGCAGGAAGACAGAATAGGAAAATTATTCTAAATAACCAG
ATCTTCATCCTCATGTGTCTGTTTTGCAGGGAATGCCTCGGCTCAGCAGGAAGGCTTCGCCGAGCCCCACCCGCAGAAGT
GTCTTTGATTAGCCCCCCCCATTCCACCTTGAGGTGCTGCTTTGGCCTCAAACCACCAATGAGATTTCTACAAATGTTTG
CCTCCATGAGGAGCCCAAAATGCCCTTCAGATCCTCCTTTTGTACTGTTTTCTGCTGTTCTGTTGACCAATCCGGAGTTT
TCTGATCCCCCCCCCCCAGTGTTGAATGGGCCATCAGACCGGCTCCACAGAAGTCCTCCGTTTGTATTTACAGAGAATC
TCAGTATTTTGAATATTTATCAGAGTCTAGTTTTAATGCAGAGATCCTTTCTCTGTACAGATGCTGCTCTCAGTCTGCTG
CCCTGTAATACACGGCGTCTGTCCTCTGAGGTTCTAGAATGTTCTGGAAGGTTCTGCTGTTCGTCTGAAGTGCCTTCACT
TTGTTTTGTGACAATAAACTTTTAATAAAGTGTAAACCCATGTGCTGGTGGTGGTTCTGATCCGTCTGTAAGGAGGAAAG
GAAAACCTTAGAGCAGCATAGGATCTTTGAAGGTCAGCATTTAGAACGGTTCTGCACTTCTTCCAGGTGCAAACCCGTCT
TTGGTCAGCTTCGTTTAGAAAAACTAAAAATCAGGTCTCTCACCTCAAAATATCATCTGAAGTCAGCTTCAAGCTGATAT
TCTGAGCAGAAAAAACAGATTTTCAGTGAACACAGAAGCTCTGAACTGATGACAGGTACAGGTCTGATTAGAACCTGGAC
CCTGCTGAGCCTCATGGTGCCGTGAGTCCAAATCCTGTCTGAATGTCATCAGAACGCCCTGCAGGTGTACGCGAACAGGC
AAACTTCCACTGGAGTCCCTCCTAAAGTCTGTTGGAGGTTGCCATGGAAACATCATTCACTTTAATGTGAGTCGCTCGGA
CTCCTCTTCAAACCTTTCTCTGACGGTTCTGGATACGTTCTCCTCAGGAGTTCACCTCCTCTGAACCTTCGCCGCTTCTC
CGTTTGAGGCTGATCACACTTGGAGAAGAAAACAGTCTTTTCTTGCGATGGAATGAAGACGAAAGCTCAGCTCAGACGTC
AGCTCAGACCTCTGTCACTTCTGATGATCAAAGTTTCTGAGAAACCTTTTTCCCCCAGAATGCATTACTGTGTTTGCTAA
ATTTGGAGGTTATTTCTGCACAAAACTTTAGATTTCAGACCATTTAAAGTTTTTCCAGCTGGAGCTGCATGACGGAGCGA
GAGCTTCACGCCTGCTGCATGACTGCTCTCCTGCAGGAACATGGTGAGAAGAAACTGGGTCAGAACCTGCAGTTAAGTCT
GGTTCTTCACCCTGAGACCAGCTCTTAAAACCTCCAAACTGACCTGTGGTGGAGTGGCGAGGTGCTGGTTCCATCTCCCA
ATCGGGACGTTGGGGTTTTGTTTGATTGGGGGGTGGGGGGTGGGGTGTGTGTCTGGAATCTGGAGATTTGGTTCCCATCC
TGTAAAACGGGACGTCGGTCTGCGAGAGCAGCTTCCGTTGGACATCCGAGTCGCGTTCAGAGTCCAGATCCTGCAGGGAC
AGAACCAGCAGCGGTTTTTGAGCTGACTCAGCAGAATGCAGTTTTCTGCTGAGTCGGGTTGGAGACCGGTGGCTGCCTCC
TGCACGGCGCTCCTCATCTGCCGCCGCCTCCTTTCTTCCCTCAGCTCTGCCTCCATGTGGCCCATCTGGGCTCGGAGTTT
GACCACTCTGCGGCAGCCGTTCCGGCGCTGCCTCCTGGATCTACCTGGGATGGATCTACCTGTTCATGACGCTTTGTATG
TTTTCTCTCTATACCAACAGCCATTGGGATAGGCTCCAGCAACCCTGACAGGGACATGGCGGGTTTGGAAAATAGACGGT
TGTTCCAACAGTAACATGGAGGGGGGTGTAAACACTTTTTGGTACCAGTCCAGTTGTTTTGGCAGAAGCGCTGGGCTCTG
ACCTCTGAACCTGCAGGTCCGTCAGTCCTTGCTGCAGGGTGGGGACGGTTCTTCCCCTTTATATCAAACAACACAAATGT
TAGAAAAAAGGTCCAACAGGAGTTTAGACTCATTAAAGGCAGGAGTGTTTTCTCTGATCGCAAATGTTGTTCTTCGTCTT
GATAAGACGATAAAATAATTGTAACACTTTCGTGGATATTTCTCACACGAATTATCAGAGAAAATCTGAGATTTTCCCAG
AAGAGGATGTACCAGAAATGTTTCCTCAAGGTCAGGCAGAAAAAAACCAGAGCCTTTCTGCTTCACTTGACATAGAGGTC
AAACTGCACCTGTTAAAGATAGCGGTTCAATAAACACTGCCAGGAGCACGCCAGCATGGCGGAGGGGCCATGATGGGGGC
TCGTTTTTTTGGGGGCTCGGGGGCCGTCAACTTGAACATCTCAAAGCAAGTGATGCCTTAAAACCTAAAGAGGTTTGAAA

CCCAGAAAAGGAATTGTTGGCCTCCCATCCGGCCTTCACAGATTCAGGTGGTTCAGGACGTCCAAAAATGAAATGTGCAG
TTGGGAAATGAAAAGACACACAAGAGTCTGCATGAATTTTAAAACCTTTTGCAAAAGGGGAGAGGTGGAAAAACACGACC
TGTTAAGCTCTGTGGCTGTCCGGTCTCTCTGTCCTCAAGGCAAAAAGGTTTTTTTTATTATCCAGTAAGGGAAGTTGAAA
ACATACAAAATACGTCATTAGAATGGTGTGTTTTTGTCTAGTCTGCATTGCAGGTGTTGTGGCTCCATAAACACTCCAGT
ACAGAAAGAAAACAGAGACCACAAGTCAAAAACAGCCTTACTGGGGACCTCGTCAGATATTATCTGCTACTGAGAGCAGG
AATCAAGCATATTTCTGCAGTACAATATACTCGCATATGTAGAATCCATTAAATGCATAATAACAAGAACATAGAAAATA
TAATAGAACCCCTGTTCACCTTAACAGAAGCAAATCTGCAAACTGGGTTCTGACTTTCAGCGTCTCACCTGGACTCACCC
TGGGTTCTGGACTGACTGATCAGGACCGCCGTTTAAGTCCAATCGCTGGATCAAACCAAGAGTGAACAAATTTGTTAGTT
ATTTTATTGTGAAGCAGTAAAACATCAAAATAGACTTTTGGTTGTGCATGGTGGTACAGTGGTTGATGCCCCAGCCTCAC
AGCTGGAAGGCCCTGATTTGAATCCCAGCTGGGTATGTGCATGCATGTGTGTCCCTGCAACAGTCTGACTATCTGCTCGC
CTCCACCCAACAGAGTAGCCGGGATAAGCTCCAGCAACCCTGTGACCCAGACGGGTCTGTGGGGTCTGGGGATGAATGGA
TGGAGTTGATTTTTAGTAGTAGATGGTTCAAAGATCCCACCTGATCTCTTCTGTGGTACTTTAGTCTGGATCCTCTGCCA
TGCCTCTTCTGACAGATTTGGACCAGAACCTCATCTCACAGGAGACTGTGTTGTGAGGCCCTCAGGATCCTCACAGGAGG
TGATGGCTCCTAACAGAAGCCCAGCTGTTCTCATGGCAGATTCAGCAGACAATTTGCTTCTCCTTTCAGTCCCATCAGCT
TCCTCTGATGCAGAGGAGGAGTTTCTCCTCTGGAGCTGTGGGTGGGAGACTGAGGTCAGATTTCACTGGAGCAAACTGCT
TTCTGGTGCTTCAAACTATTTTTATTCAATGAAATGTATATATATATATTTCTCTGCTGCAGATGATGAGCTGACCAGCT
GATTAGCCGACTGTAAAGCCCTGTGAAAGTTGTACCTCCTCAGAAACTCCAAAACTGTTGTTCCCGTCTCCAAACCTCTC
GTGTTCCAGCAGGTTCTTCATCTCCAGGCCGTGAAGCTCATCTCTGTGTTTCTTCTCCTGTGCTTCAGCTGCTTCTGCAG
GGAGGCAGTGGTGGAGGTCAGCTGGTCTTTGTGCTCCAGCTGCTCCTCTGTGAGGACAGGAGTCTCTTTCAGCCTGTTGT
GTCACACCGTCTTCTCTGGAGAAACGACAGCCAGGTCAGCGACGTTCAATCTGATGTGTCAAAAGATCTTAAAATGCTCA
GGATTTTCAGAAGTAGCAGATGCAGAAGATCTTACTTTTTTTTTTTTTTTTTACCAAACTTTCATCCGCCTACAGGGAGA
AGGTCTCACCAAGCTCCCTGTTTCTCTGCGTGAGTTCCTGGATTTGCTCTTGGAGCTCCAGACTGAGCTGCAACTGCTGC
AGAGCGTCTCTGTCGTTACCTGCAGCCTCCTGCTGAAGCTCCAGCTGCTCCGACAGCGCCTCCACCCGCTCCTTCTCCTC
CAGCAGGGAGCGATTCAGATGCTCGATGATGTCGTTCTTGTCCTTCTCCTCCTCCTTCTCCTCCTTCAGCTGAAATC
TGTAGGAGGATGTTAAAAAAACACGTCTGAATATGTACGTAACATTTAGGTTTATTTACATGATGCTATGTTAAACTAAC
AAAAAGAGAAAATAATAACTCATTCAAAGTCACAGTCTGTTCCATTGACTGTATCTAAGCACTGGACTGAGTGACGTCCT
CAAATGGTTTCTTCTGCCTTCAACCAAATGAAGTCAATTCTGTTGAAATTGCTTTCAGATACAGACGCTGGGGGCTAGAC
GTCGTGAACATGCTGTGAATTGGTTGGGCTTAACGTTTCTAAGGCAACCACTCTGGGCAATCAGGGGTGAGCTTATTAGA
AGCCCACACCCCTACCTCTTGAAAATAGGCTACATCAAATCTATCAAACACTTTGAACGTTGGAAGTGGGGGCCAACCAT
TGACTGTATGTGAGAACTGGACTGAGTGACTCCTCCCCCCTGGGGCTCCAAACAGGAAGTGGTTCCAAAATGCCCAAATC
CCACAGACTTCTATAGAGAAACTACAGCTGTTCCTCAGTCATTCAGAAGAATCGTTCTGGATCTGCAACCATCATCCTGA
TAATTCTCTGTTTTTTCAGGATATTTTCTCTGTAGTTCAAGTTGTTTATGTTAGAAACTGACCAATTAGATGCCTCAATA
AAAGTAGGTGGAGCCTGCTGCCCCCACATCCAACGTTCAAAACAATTTATAGAGTTGGTGTAGCCTGCTTTGAAGTGGTA
GGGGTGTGGCTTTCCAACAAGCTCACTCCTAATAGGTGAGAATTGTTGCTATAGAAACATGAACTCAGACAGACTCGGAC
TGTTTAGTTAAAGCTTTTACCAATCATACCTCTCATGCCATGTTATCATCGTCACATAAACAGTAGGATGCGGTTGGGAT
AATAGACAGTAATGGTCCGGCATCTCATTTCTGACACCATATGAGCACATTATAAAACTGTAGCGACCTGGGGGTTAGCC
CCGCCTTCAGCCCCTAAGATTCGTGGGAAGTGGGGAGCTTAGGGTGAAATCTTGAGTCAGAGGGCTGGAAGCTGAAGTGA
CCCTCAATGCTGTTTGGGCTGTATGTGTGCTTGAATAAACGGGCTTGATGCTCAAAAGGAGACATTGCCTTTCTTCCTGT
TTATTTCTGCCTGAGACTGTCCGGGGTCGTGCGGTGTATGGGGCACGGACAGCTCTCCAATGAAGCCATCTAAATAGCAG
TGTGGCTTTTGCTTCCCCACTAAACTGACGGTCCGGCTGGATCGTTACATTACTTAGATTTTTACTTATTCGCGTTTAGA
CAACGTTTATGTTGTAATCAATTGTGTGTTTTGTGAACTAAGAATATGGCAGCCAAGAGACTTCCTGCCAGTGACTTCCG
GTCTCTCCGGCGTGTGGCTCAATGAGTTATATCACTAGAGGAGACACGCCCGTTTTAGAAGCCGGGCCAACGGTGGCGGA

GCGCGACACTATCTTGGTGAGGTCGACGATGCCCACATGACAATGATTTCTATTGCTTTTAGAGGTGTCTAAGTAGCTTT
TATATACTATATATATTTTTAAATTTATATATATTTACACATATATGTAAATATATTTATATATATAATATATATATAGA
TACATCATTTGTTGTTGTTAAAGAAGAGAAACAGTCAGCCTTAAAAGCTCAAACTTTAATGAAAATGCATTGTTGCAGGT
CCTCTTACAAATAAAAAAATTACTACAAAGAATTTTGAAAAAAATAATTTGTAAACATTTAAGAGTTTTAGACCTCTTTG
ACATGTAATTTAAATTATTTAGAAAAACCCAACAATAAGTCAATAATAATAATAATAATAATAATAGTAAATCAATAGTA
GGTCAATACAAACAATAAATAATAACAATACATAAATAATAAGTCAATAAATAATAATAAAAATAAGTCTATTAATAATA
ATAATAATAATAATAATAATAATAATAATAATAATAATTTTAATAAAAATAAAATAAATATTCAAAAGAAGTCCAAAAGT
GAACAATAGATTTTCCCTTAAAAAAACAACAAAAAAGAAAAACAATAGATTTATACTGATTCATGTGAGTGATCAATTAG
TGTAACACAGATGTTATGGTGTCGGGTTTCAAGCAAACAGTCTGGCCTCTTATCTAAATGTGTTTCCACAAATGTTCTGA
ACATAGCCAGACTGTATTACTAGTTGGTACCACAGTGATCCATCTGGGTTAGGTCTTTTTATTGCTAAGATCCACTTTTT
CCTTAAGTCTGAATCCAAATTCAAATTCAAATTCAAATTTTATTTGTCACACACACAATCATACAGCGTACAATGTGCAG
TGAAATGCTTAGGCGACCGCCCGCGACCTCAAACAAGAAAGAATATCAATAGGAATAGGAAGATAAATATAAAATATAGG
AATATACAAATATAAAAACATAAAAACAGAATGTCCGAATGTGTGCAAATATGCTCTAAAAGTGACATGTGAAAGTGGCT
TGTGTGTATAATGTCCATGATTACTGGTTAAGAGTCCGTATCGCCTGCGGGAAGAAGCTCCTCCTCAGTCTCTCTGTGTT
AGCTTTCAGAGAGCGGAATCGCTTTCCTGACCGCAACAGCGAGAACAGTCCGTTGTCAGGGTGGCTGAGGTCCTTTGTTA
TCTTCCTGGCCCTGGTCCAGCACCGCCTGCCGTAGATTGAGTGCAGGTCAGGGAGCTCGGTGTGGATGATGCGCTCAGCT
GAACGCACCACCCTTTGGAGAGCTCGTCTGTCCTGCATGGTGCTGTTCCCGAACCAGGTTGTGATGTTTCCCGTCAGGAT
GCTCTCTATGGTGCAAGAGTAAAAGTTGCTAAGCACCACAGAGGGCAGTCTAAAGTCTCTCAGGCGTCTAAGATGGTAAA
GACGCTGCCGGGCCTTTTTCACCACGGTGTTGATGTGACAGGACCATGACAGGTCCTGTGTGATGTGGACACCGAGGTAT
CGGAAGCTGTTCACTCTCTCCACTGGGCTCTTGTCGATGACGGGGGTCTGGTAGTTCCTCTTCTGCTTTGTGGCGAAGTC
CACTATCAACTCCTTTGTCTTGCTGACGTTTAGGAGGAGATTGTTCCTCTCACACCAGTTTTCCAGATGTCCAATCTCCT
TCAGGTAGGCCGTCTCATCATTATCAGTGATCAGGCCCACCACAACGGTGTCGTCAGCGAATTTGATGATGGTGGTGGAG
TTGGTGGACGCCACGCAGTCGTACGTGTACAGTGAGTACAGTAGAGGGCTCAGAACACAACCCTGAGGATCCCAGAATCC
ATTGGAAATCTGGAAGAAAAGTTCAGTCTCTGAGTCAGAAATGTATAAATCTATATTACTATAATAGCCTAGTATGCTGT
ATACAGGTTAAGCAAAATAAAATGTACATTTATGTCGGCAGCAAATTATACGTACATCATGAAGCATGTTGATAACAGCC
CTGCAGCATCATGCATGTCAACATGTGTATGATTAATGAAGCCTCTGCAGCATTAAAAGAAAAAAAATTATAGTACTTAC
CTGTGAAATGATATACCTTCCTCCTTCATGGACTCAGTTCTCTGATTTTTGGAAGTTGAAACCCGCACAATGAACTGGTA
TTCTGCAAAAAATGTATTCACATGCATACACTGCAGCAGTAACCTGACCTCACCAAGATGGCGGTGATCTCCTCCCATGA
GGAACCACGTGATGTCTCCTCTAGCGATATAACTCATTGGTGTGGCTGATTCCAGCTAATGACGAAACAAAAAGGGGGGG
GGGGGGGGAGTCGGAGCCAAGCGGGTACAACATTCTGAAAGACTGTTCTCCGGTAAATTGACGCACAGCATCTGGCTTCA
CCGTTGGAGGATTTAACGAAACTCTGCAACTTACTGGAAGGTATTCCCTTTGACCGGAAAGTCAGACGAATTC
