# Supplementary material for: Transcriptional Rewiring of the Sex Determining dmrt1 Gene Duplicate by Transposable Elements
Source: PLoS Genet. 2010 Feb 12;6(2):e1000844. doi: 10.1371/journal.pgen.1000844 (PMC2820524; doi:10.1371/journal.pgen.1000844)
Supplement: Figure S3 — Putative THAP domain in the Izanagi element. (A) Structure of the Izanagi element. Pink boxes indicate the three putative exons constituting the THAP domain are. The insertion of repeat 2 in the dmrt1bY promoter splitting repeat 1 into repeat 1b is indicated by the arrow. Note that the Izanagi element is shown here in reverse complement compared to the dmrt1bY promoter. (B) Alignment of the putative THAP protein domain from the Izanagi element consensus sequence with the THAP domain from the PFAM database. Identical essential residues are yellow shaded; other identical residues are blue shaded. + indicates similar residues. (1.01 MB PDF) [file pgen.1000844.s003.pdf]

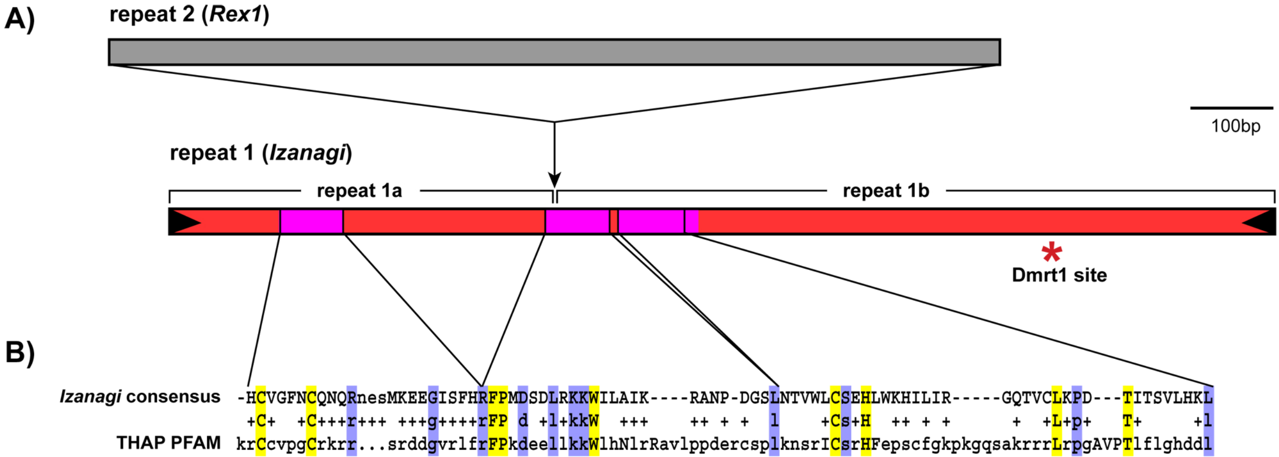
A)
repeat 2 (Rex1)
100bp
repeat 1 (Izanagi)
repeat 1a
repeat 1b
Dmrt1 site
B)
Izanagi consensus -HCVGFNCQNQRnesMKEEGISFHRFPMDSDLRKKWILAIK----RANP-DGSLNTVWLCSEHLWKHILIR-----GQTVCLKPD---TITSVLHKL
+C+ +C+++r+++ +++g++++rFP d +l+kkW +++ ++ + + l +Cs+H ++ + + + +L+p+ T+ +l
THAP PFAM krCcvpgCrkrr...srddgvrlfrFPkdeellkkWlhNlrRavlppdercsplknsrICsrHFepscfgkpkgqsakrrrLrpgAVPTlflghddl
