# Supplementary material for: Transcriptional Rewiring of the Sex Determining dmrt1 Gene Duplicate by Transposable Elements
Source: PLoS Genet. 2010 Feb 12;6(2):e1000844. doi: 10.1371/journal.pgen.1000844 (PMC2820524; doi:10.1371/journal.pgen.1000844)
Supplement: Figure S5 — Activity of dmrt1bY promoter deletion constructs in different cell lines. (A–C) Various 5′-deletions mutants (3 Kb and 6 Kb) from pBSII-ISceI::9 Kb dmrt1bY prom::GLuc plasmid were transfected either into Mouse TM4 Sertoli, Xiphophorus embryonic epithelial A2 or medaka HN2 fibroblast like cells. Transfections were repeated three times; error bars represent the standard errors of the means. (0.62 MB PDF) [file pgen.1000844.s005.pdf]

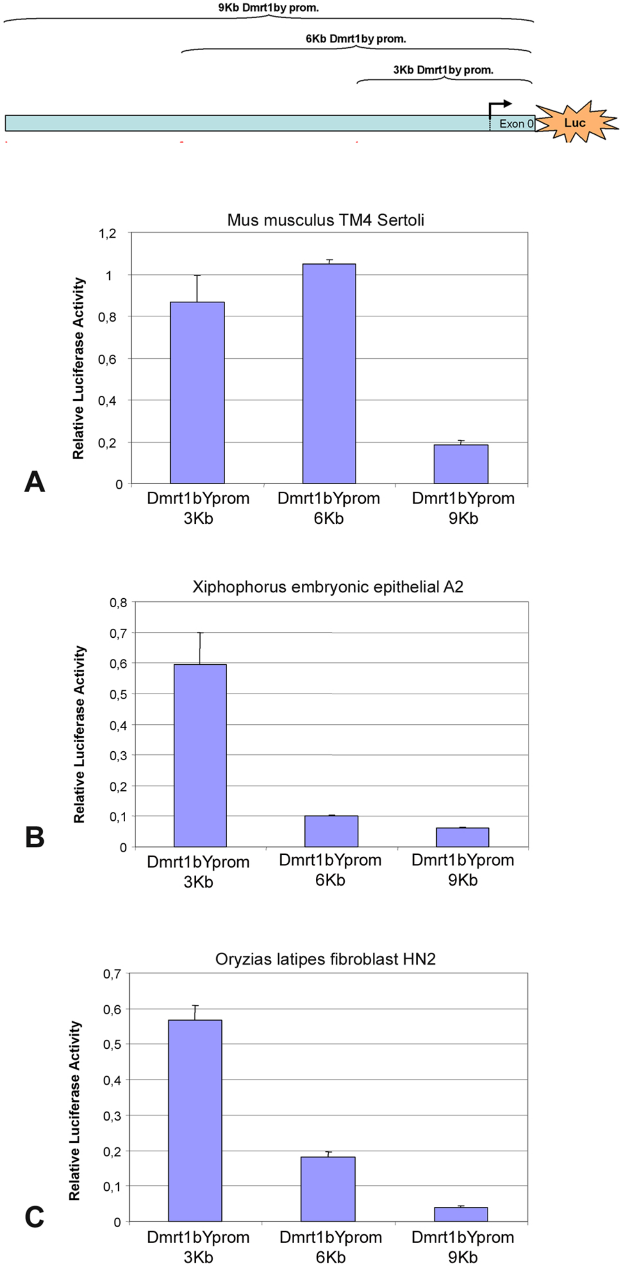
9Kb Dmrt1by prom.
6Kb Dmrt1by prom.
3Kb Dmrt1by prom.
Exon 0
Luc
A
Mus musculus TM4 Sertoli
Relative Luciferase Activity
1,2
1
0,8
0,6
0,4
0,2
0
Dmrt1bYprom 3Kb
Dmrt1bYprom 6Kb
Dmrt1bYprom 9Kb
B
Xiphophorus embryonic epithelial A2
Relative Luciferase Activity
0,8
0,7
0,6
0,5
0,4
0,3
0,2
0,1
0
Dmrt1bYprom 3Kb
Dmrt1bYprom 6Kb
Dmrt1bYprom 9Kb
C
Oryzias latipes fibroblast HN2
Relative Luciferase Activity
0,7
0,6
0,5
0,4
0,3
0,2
0,1
0
Dmrt1bYprom 3Kb
Dmrt1bYprom 6Kb
Dmrt1bYprom 9Kb
