# Supplementary material for: Transcriptional Rewiring of the Sex Determining dmrt1 Gene Duplicate by Transposable Elements
Source: PLoS Genet. 2010 Feb 12;6(2):e1000844. doi: 10.1371/journal.pgen.1000844 (PMC2820524; doi:10.1371/journal.pgen.1000844)
Supplement: Table S1 — Location and adjacent genes of repeat 1 elements containing Dmrt1 binding sites in the medaka genome. (0.06 MB DOC) [file pgen.1000844.s008.doc]

# Supplementary Table 1:

# Location and adjacent genes of repeat 1 elements containing Dmrt1 binding sites in the medaka genome. Many elements have repeat 1 terminal inverted repeats (TIR) and 8bp target site duplications (TSD), others are truncated versions.

| **chr** | **scaf** | **ctg** | **TIR** | **TSD** | **within gene…** | | **5’ gene** | | **3’ gene** | |
| --- | --- | --- | --- | --- | --- | --- | --- | --- | --- | --- |
| 18 | 158 | 62358 | x | - | UTOLAPRE05100116165 | nrxn2 | ENSORLG00000011152 | itsn1 | UTOLAPRE05100106184 | pdss2 |
| 2 | 421 | 94528 | x | - | ENSORLESTG00000008579 | unknown | UTOLAPRE05100108644 | helitron | - | - |
| 10 | 1187 | 112105 | x | - | - | - | UTOLAPRE05100117612 | cd2 | ENSORLG00000004786 | pdgfrb2 |
| 2 | 6 | 6090 | x | - | - | - | ENSORLG00000004467 | adcyap1b | ENSORLG00000004478 | gabrg3 |
| 2 | 6 | 6161 | x | x | ENSORLG00000004601 | unknown | ENSORLG00000004598 | spry2 | ENSORLG00000004606 | slitrk1 |
| ? | 681 | 104338 | x | x | - | - | - | - | UTOLAPRE05100109284 | unknown |
| ? | 689 | 104555 | - | - | - | - | - | - | - | - |
| ? | 617 | 102588 | x | - | - | - | UTOLAPRE05100119300 | unknown | UTOLAPRE05100119298 | lrp4 |
| 14 | 14 | 12353 | x | - | - | - | ENSORLG00000015350 | clip2 | ENSORLG00000015354 | cldn2 |
| ? | 84 | 43849 | x | x | - | - | ENSORLG00000019785 | c8orf30a | UTOLAPRE05100114491 | rbbp5 |
| ? | 1230 | 112458 | x | - | - | - | - | - | - | - |
| ? | 837 | 107543 | x | x | - | - | UTOLAPRE05100109411 | thap1 | - | - |
| 10 | 326 | 87316 | x | - | - | - | ENSORLG00000008548 | slitrk4 | UTOLAPRE05100106149 | unknown |
| 3 | 75 | 40801 | x | x | - | - | ENSORLG00000011067 | adpgk | ENSORLG00000011080 | ctsb |
| ? | 1786 | 116942 | x | - | - | - | - | - | - | - |
| 17 | 219 | 73734 | x | x | - | - | ENSORLG00000017872 | adcyap1 | ENSORLG00000017875 | yes1 |
| ? | 603 | 102191 | x | - | - | - | ENSORLG00000019970 | zinc finger | - | - |
| ? | 261 | 79835 | x | x | - | - | ENSORLG00000019715 | gcm2 | ENSORLG00000019726 | arhgap28 |
| ? | 615 | 102552 | x | x | - | - | ENSORLG00000019409 | MHCIIa | - | - |
| 6 | 61 | 35810/1 | x | x | - | - | ENSORLG00000002113 | RT | ENSORLG00000002126 | avpr1a |
| 6 | 411 | 93931/2 | x | x | ENSORLG00000000223 | tph2 | ENSORLG00000000184 | tbc1d15 | ENSORLG00000000232 | mphosph6 |
| ? | 1363 | 113652 | x | x | - | - | - | - | ENSORLG00000020433 | nubp1 |
| ? | 2260 | 119798 | x | - | - | - | - | - | - | - |
| 1 | 331 | 87797/8 | x | - | - | - | ENSORLG00000014321 | chrnb6 | UTOLAPRE05100108110 | unknown |
| 7 | 21 | 17172 | x | x | - | - | ENSORLG00000002861 | arih2 | ENSORLG00000002861 | impdh2 |
| 19 | 53 | 32543/4 | x | - | - | - | ENSORLG00000013155 | glpr2 | ENSORLG00000013160 | c17orf48 |
| 10 | 18 | 15290 | x | - | - | - | ENSORLG00000008496 | rps6ka6 | UTOLAPRE05100101558 | unknown |
| 18 | 386 | 92203 | x | - | - | - | UTOLAPRE05100108465 | btn2a1 | UTOLAPRE05100108464 | fcrl5 |
