# Supplementary material for: Transcriptional Rewiring of the Sex Determining dmrt1 Gene Duplicate by Transposable Elements
Source: PLoS Genet. 2010 Feb 12;6(2):e1000844. doi: 10.1371/journal.pgen.1000844 (PMC2820524; doi:10.1371/journal.pgen.1000844)
Supplement: Text S1 — Izanagi and Izanami: Creators of Japan. (6.88 MB PDF) [file pgen.1000844.s009.pdf]

Izanagi and Izanami: Creators of Japan

Izanagi no Mikoto ("the male who invites") and *Izanami no Mikoto* ("the female who invites") are two of the primary kami found in ancient Shinto mythology. The divine siblings are the deities of the terrestrial creation myth, whereby the lands and all the creatures that inhabit them came into being. Specifically, they are honored as the originators of the islands of Japan.

The new transposable element described in this paper was named *Izanagi* according to the fact that it is essential for male development and possibly contributed to create *Oryzias latipes* species. For more information about Izanagi and Izanami please refer to: https://www.uwec.edu/philrel/shimbutsudo/izanami_izanagi.html

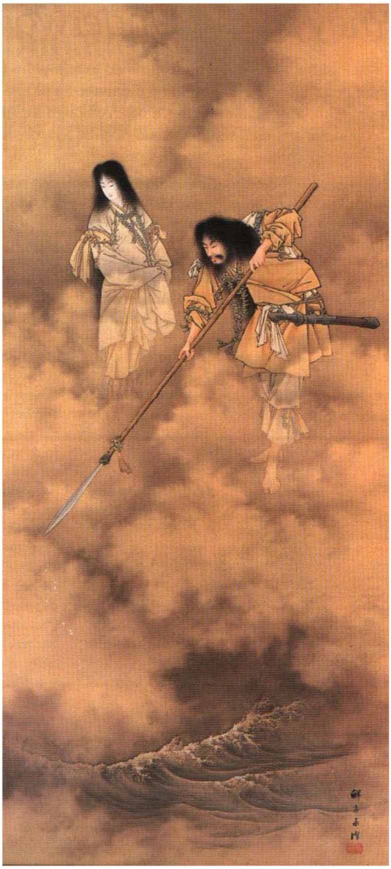

"After settling down in Onogoro island, Izanagi invited Izanami to describe how her body was formed. She said, "My body in its thriving grows, but there is one part that does not grow together." Izanagi replied, "My body in its thriving also grows, but there is one part that grows in excess. Therefore, would it not seem proper that I should introduce the part of my body in excess into the part of your body that does not grow together, and so procreate territories?" Izanami said, "It would be well" (*Kojiki 20; Nihongi 14)*. Hence, they were the first to procreate sexually".

Kobayashi Eitaku, *Izanagi and Izanami*, c. 1885.
http://commons.wikimedia.org/wiki/File:Kobayashi_Izanami_and_izanagi.jpg
